# Blood T Helper Memory Cells: A Tool for Studying Skin Inflammation in HS?

**DOI:** 10.3390/ijms24108854

**Published:** 2023-05-16

**Authors:** Katrin Witte, Sylke Schneider-Burrus, Gabriela Salinas, Rotraut Mössner, Kamran Ghoreschi, Kerstin Wolk, Robert Sabat

**Affiliations:** 1 Psoriasis Research and Treatment Center, Charité—Universitätsmedizin Berlin, Corporate Member of Freie Universität Berlin and Humboldt-Universität zu Berlin, 10117 Berlin, Germany; 2 Interdisciplinary Group of Molecular Immunopathology, Dermatology/Medical Immunology, Charité—Universitätsmedizin Berlin, Corporate Member of Freie Universität Berlin and Humboldt-Universität zu Berlin, 10117 Berlin, Germany; 3 Berlin Institute of Health Center for Regenerative Therapies (BCRT), Charité—Universitätsmedizin Berlin, Corporate Member of Freie Universität Berlin and Humboldt-Universität zu Berlin, 13353 Berlin, Germany; 4 Center for Dermatosurgery, Havelklinik Berlin, 13595 Berlin, Germany; 5 NGS-Integrative Genomics Core Unit, Institute of Human Genetics, University Medical Center Göttingen, 37073 Göttingen, Germany; 6 Department of Dermatology, Georg-August-University Goettingen, 37073 Goettingen, Germany; 7 Department of Dermatology, Venereology and Allergology, Charité—Universitätsmedizin Berlin, Corporate Member of Freie Universität Berlin and Humboldt-Universität zu Berlin, 10117 Berlin, Germany

**Keywords:** acne inversa, biomarker, CD4 memory T cells, cytochrome C oxidase, IL-17, neurodegenerative disorder, oxidative phosphorylation, proteasome, respiratory chain complex, transcriptome

## Abstract

Hidradenitis suppurativa (HS) is an inflammatory skin disease characterized by painful lesions on intertriginous body areas such as the axillary, inguinal, and perianal sites. Given the limited treatment options for HS, expanding our knowledge of its pathogenetic mechanisms is a prerequisite for novel therapeutic developments. T cells are assumed to play a crucial role in HS pathogenesis. However, it is currently unknown whether blood T cells show specific molecular alterations in HS. To address this, we studied the molecular profile of CD4^+^ memory T (Th_mem_) cells purified from the blood of patients with HS and matched healthy participants. About 2.0% and 1.9% of protein-coding transcripts were found to be up- and down-regulated in blood HS Th_mem_ cells, respectively. These differentially expressed transcripts (DETs) are known to be involved in nucleoside triphosphate/nucleotide metabolic processes, mitochondrion organization, and oxidative phosphorylation. The detected down-regulation of transcripts involved in oxidative phosphorylation suggest a metabolic shift of HS Th_mem_ cells towards glycolysis. The inclusion of transcriptome data from skin from HS patients and healthy participants in the analyses revealed that in HS skin lesions, the expression pattern of transcripts identified as DETs in blood HS Th_mem_ cells was very similar to the expression pattern of the totality of protein-coding transcripts. Furthermore, there was no significant association between the extent of the expressional changes in the DETs of blood HS Th_mem_ cells and the extent of the expressional changes in these transcripts in HS skin lesions compared to healthy donor skin. Additionally, a gene ontology enrichment analysis did not demonstrate any association of the DETs of blood HS Th_mem_ cells with skin disorders. Instead, there were associations with different neurological diseases, non-alcoholic fatty liver disease, and thermogenesis. The levels of most DETs linked to neurological diseases showed a positive correlation to each other, suggesting common regulatory mechanisms. In summary, the transcriptomic changes in blood Th_mem_ cells observed in patients with manifest cutaneous HS lesions do not appear to be characteristic of the molecular changes in the skin. Instead, they could be useful for studying comorbidities and identifying corresponding blood biomarkers in these patients.

## 1. Introduction

Hidradenitis suppurativa (HS; also referred to as acne inversa) is a chronic inflammatory disease with persistence of characteristic skin alterations [1]. These include painful, deep-seated, inflamed nodules and abscesses that occur in early adulthood in the axillary, inguinal, and perianal skin areas. As the disease progresses, pus-draining sinus tracts develop and the normal skin architecture is destroyed, resulting in scars and contractures [1]. The prevalence of HS in Europe, North America, and Australia is about 0.5–2% [1,2]. In these countries, HS affects both sexes with similar frequencies; however, men more often show lesions in the axillary area and women in the inguinal area [3,4,5]. Patients with HS frequently suffer from anxiety, limitations in many areas of life, including their sexual and professional lives, and stigmatization [6,7,8,9]. Furthermore, patients with HS are often affected by metabolic syndrome, nonalcoholic fatty liver disease, atherosclerosis, spondyloarthritis, inflammatory bowel disease, schizophrenia, and bipolar disorders [10,11,12,13,14,15,16,17,18]. The importance of HS arises not only from its profound negative impact on the health as well as the social and professional lives of patients but also from its strong negative socio-economic impact on society. The latter mainly results from the enormous loss of the gross value added, which was estimated at EUR 13 billion per year for Germany [19].

The treatment options for HS are currently very limited [1]. They include the surgical removal of the altered skin areas, the long-term systemic administration of antibiotics, and administration of the anti-TNF-α antibody adalimumab [1]. Satisfactory, long-lasting results are not currently achieved in most patients treated in this way [6,20]. This makes the search for new treatment concepts imperative [21,22,23,24,25,26]. However, a thorough understanding of HS’s pathogenesis is necessary for the development of targeted, highly effective therapeutics.

The etiopathogenesis of HS is only partially understood [1]. Genetic predisposition, smoking, and obesity are important factors that lead to the onset of HS [27,28]. An attenuated production of antibacterial proteins seems to facilitate the persistence of specific bacteria in HS skin lesions [29,30]. Bacterial products and components of damaged skin cells might be responsible for the permanent stimulation of immune cells, leading to the attraction of different immune cell types from the blood into the skin and their activation there [27]. These processes are reflected at the molecular and clinical levels by the massive production of cytokines and the recurring development of nodules and abscesses with pus discharge, respectively. In fact, analyses of HS lesions demonstrated strongly elevated expression levels of several immune mediators [29,31,32,33,34,35,36,37,38,39]. Beyond cytokines, activated fibroblasts and neutrophilic granulocytes secrete matrix metalloproteinases, which mediate the tissue destruction frequently observed in HS [35,37]. The results of placebo-controlled clinical trials imply a relevant role of IL-17A in the processes underlying the persistence of skin alterations in HS [40,41]. IL-17 is a cytokine that is mostly produced in humans by a specific CD4^+^ T memory (Th_mem_) cell population [42,43]. However, investigations of Th_mem_ cells in HS are rare [44,45,46]. Therefore, we analyzed blood Th_mem_ cells from patients with HS and control participants in this project.

## 2. Results and Discussion

To gain insight into the role of Th_mem_ cells in HS pathogenesis, we aimed to characterize the molecular profile of these cells. Therefore, we used RNA sequencing to analyze the transcriptome of Th_mem_ cells purified from the blood of 15 patients with HS in comparison to healthy participants (n = 13) matched for age, gender, and smoking habit (Table 1).

**Figure 1:**
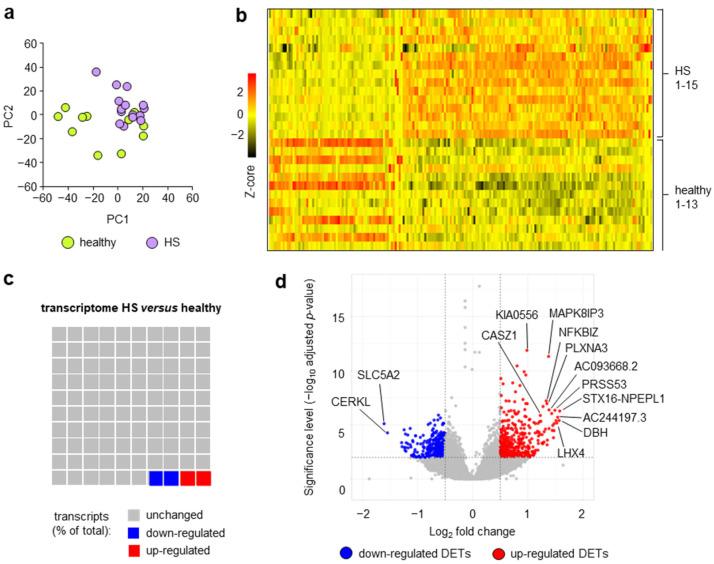
Blood Th_mem_ cells show HS-specific transcriptome alterations. Th_mem_ cells were isolated from the blood of patients with HS and healthy participants and subjected to transcriptome analysis. (**a**) Data obtained from principal component analysis (top 1000 regulated transcripts) of transcripts of Th_mem_ cells of patients with HS compared to those of healthy participants are provided as PC1 plotted against PC2. (**b**) Differentially expressed transcripts (DETs; cutoff: adjusted *p* < 0.01; log_2_ fold change 0.5/−0.5) of blood Th_mem_ cells (HS vs. healthy; predefined sample order) are presented as a heatmap based on Z-scores. (**c**) Proportion of DETs of blood HS Th_mem_ cells in total protein coding transcripts are presented as waffle chart. (**d**) Log_2_ of fold-change values of transcripts of blood HS Th_mem_ cells were plotted against their significance values and are given as volcano plot. DETs with significantly (cutoff *p* < 0.01) increased (red) or decreased (blue) expression are indicated. Gene names of selected transcripts are presented. HS, hidradenitis suppurativa, DETs, differentially expressed transcripts.

A principal component analysis (PCA) of the transcriptome data revealed molecular differences between the Th_mem_ cells from patients with HS and healthy participants, as obvious by a sub-clustering of cells from both cohorts (Figure 1a). Importantly, as shown in Figure 1b, a Z-score analysis disclosed that the molecular differences between the HS and healthy donor Th_mem_ cells were accompanied by a substantial similarity in the gene expression pattern of individual samples among each cohort.

Among 19,890 protein-coding transcripts, 772 transcripts (3.88%) were found to be differentially expressed in HS Th_mem_ cells compared to healthy donor Th_mem_ cells (Figure 1c; Table S1). Among these 772 differentially expressed transcripts (DETs), 393 (1.98%) were up-regulated and 379 (1.90%) were down-regulated (Figure 1c). To the best of our knowledge, there are no data published on blood Th_mem_ cells from patients with other common chronic inflammatory skin diseases such as psoriasis or atopic dermatitis for comparison.

Interestingly, the DETs most profoundly regulated in blood HS Th_mem_ cells (Figure 1d, Table 2 and Table 3) comprise products of genes not previously mentioned in the context of HS. Importantly, there was no significant difference in the expression of IL-17A (*p* = 0.35), IL-17F (*p* = 0.95), or IL-26 (*p* = 0.65) between HS and healthy donor Th_mem_ cells. This implies that either blood Th_mem_ cells do not significantly infiltrate established skin lesions of HS patients or that blood Th_mem_ cells obtain their specific pathogenic profile only after having infiltrated the skin.

**Figure 2:**
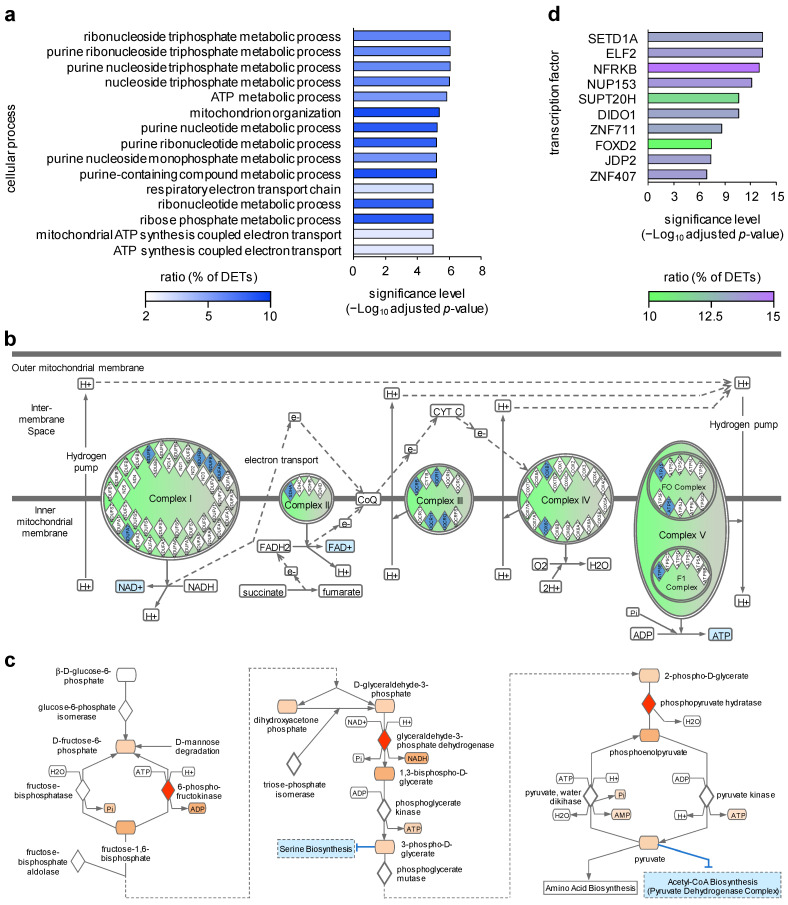
DETs of blood Th_mem_ cells of HS patients are associated with specific intracellular processes. DETs of blood HS Th_mem_ cells were determined as described in Figure 1b. (**a**) DETs of blood HS Th_mem_ cells were tested for overrepresentation of intracellular process. (**b**,**c**) Visualization of DETs of blood HS Th_mem_ cells involved in oxidative phosphorylation (**b**) and glycolysis (**c**). Up-regulated (red) and down-regulated (blue) DETs are indicated. (**d**) DETs of blood HS Th_mem_ cells were analyzed for the presence of potential master regulatory transcription factors. HS, hidradenitis suppurativa, DETs, differentially expressed transcripts.

In the next step, we investigated whether the molecular alterations observed in HS Th_mem_ cells were associated with specific cellular processes and performed a gene ontology (GO) term enrichment analysis. The identified DETs were annotated to 86 GO biological process terms. The top 15 enriched cellular processes included purine nucleotide metabolic processes, nucleoside triphosphate metabolic processes, mitochondrion organization, and respiratory chain oxidative phosphorylation (Figure 2a). This might suggest alterations in the oxidative phosphorylation, ATP synthesis, RNA transcription, DNA replication, and signal transduction in these cells. Given the fact that transcripts involved in oxidative phosphorylation were found to be down-regulated in this analysis (Figure 2b), a more pronounced shift towards glycolysis-dependent energy production can be assumed (Figure 2c) for HS Th_mem_ cells compared to healthy donor Th_mem_ cells. Glycolysis has been shown to be enhanced during the activation of naïve CD4^+^ T cells and the differentiation of these cells into CD4^+^ effector/memory subsets such as Th17 cells. However, permanent glycolysis was associated with an exhausted phenotype of CD4^+^ T cells and the occurrence of autoimmune diseases [47,48,49]. Therefore, our data may indicate that the blood HS Th_mem_ cells have a long history of activation and/or have re-migrated into the blood from the chronically inflamed tissues. The second possibility is particularly interesting but speculative and should be investigated in further studies.

In order to identify potential master regulators of the DETs of blood HS Th_mem_ cells, we performed an overrepresentation analysis of transcription factor target gene sets using the C3 gene set collection of the Molecular Signature Database. This analysis revealed NFRKB (nuclear factor related to kappa-B binding protein) as the master regulator responsible for the regulation of the largest proportion of the DETs of blood HS Th_mem_ cells (Figure 2d). It was suggested that NFRKB is a telomere-associated protein and might influence cell proliferation, but the biological function of this transcription factor is still not well understood [50]. In addition to NFRKB, SETD1A (SET domain containing 1A) was found to potentially regulate the second largest group of DETs identified for blood Th_mem_ cells (Figure 2d). As member of the SET1/MLL family methyltransferases, SETD1A was described as influencing chromatin conformation by modulating the methylation of histone H3 at lysine 4 (H3K4me3), resulting in transcriptional activation of the affected genes [51]. Interestingly, it was reported that mutations in SETD1A substantially increase the risk for the development of schizophrenia [51,52].

**Figure 3:**
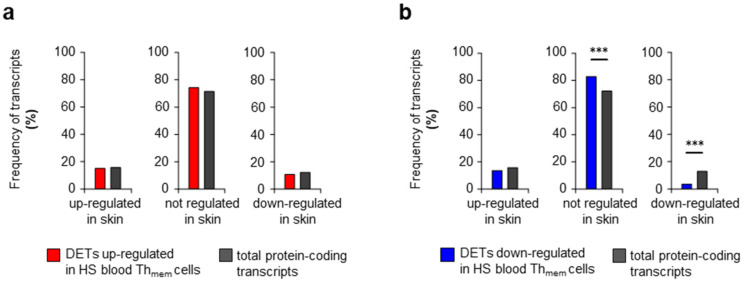
In HS skin lesions, the transcripts corresponding to the up- and down-regulated DETs of blood HS Th_mem_ cells are not more frequently up- and down-regulated than the totality of lesional protein-encoding transcripts. DETs of blood HS Th_mem_ cells were determined as described in Figure 1b. The frequencies of transcripts corresponding to up-regulated (**a**) and down-regulated (**b**) DETs of blood HS Th_mem_ cells that were up- or down-regulated or not-regulated (cutoff: adjusted *p* < 0.01; log_2_ fold change 0.5/−0.5) in HS skin lesions compared to healthy donor skin were determined based on a previously published data set [35,38]. For comparison, the proportions of up- or down-regulated or not regulated (cutoff: adjusted *p* < 0.01; log_2_ fold change 0.5/−0.5) transcripts among the totality of protein coding-transcripts in HS skin lesions compared to healthy donor skin are shown. Differences in the frequencies of up-/down-/not-regulated transcripts corresponding to DETs of blood HS Th_mem_ cells compared to the totality of protein-coding transcripts in HS lesions were tested using the *X*_2_ test (*** *p* < 0.001). HS, hidradenitis suppurativa, DETs, differentially expressed transcripts.

Next, we asked whether the transcriptomic signature of blood HS Th_mem_ cells can be found in the lesional skin of HS patients. For these analyses, we used our recently published transcriptome data of the skin samples taken from the axillary region of HS patients and from equal sites of healthy participants [35,38]. First, we tested whether individual DETs detected in blood HS Th_mem_ cells were significantly up-regulated, down-regulated, or unchanged in the transcriptome of HS lesions compared to healthy donor skin. Surprisingly, most transcripts up-regulated or down-regulated in blood HS Th_mem_ cells were not found to be regulated in the same direction in HS skin lesions (Figure 3 or Table S2). In fact, the majority of transcripts corresponding to the DETs of blood HS Th_mem_ cells (74.9% of up-regulated DETs; 83.2% of down-regulated DETs) were unregulated in HS skin lesions. Moreover, the proportions of transcripts up-regulated in HS lesions did differ between the following groups: transcripts up-regulated in blood HS Th_mem_ cells (14.6%), transcripts down-regulated in blood HS Th_mem_ cells (13.1%), and the totality of transcripts in HS skin (15.6%) (Figure 3 or Table S2). Even more surprising was that the proportion of transcripts down-regulated in HS lesions among the down-regulated transcripts in blood HS Th_mem_ cells (3.6%) was significantly smaller than among all transcripts (12.5%) (Figure 3 or Table S2).

**Figure 4:**
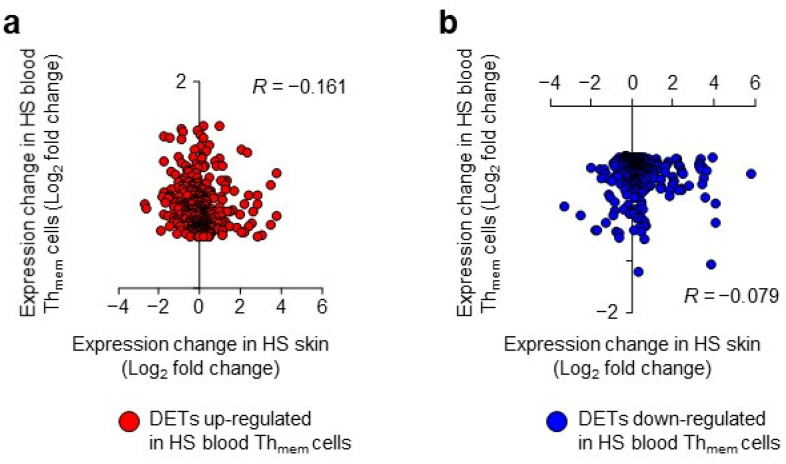
The DETs of blood HS Th_mem_ cells show no association with HS skin transcriptomic signature. DETs of blood HS Th_mem_ cells were determined as described in Figure 1b. For the transcripts corresponding to these DETs of blood HS Th_mem_ cells, the expressional changes in HS skin lesions versus corresponding samples from healthy participants were calculated. Expressional changes (log_2_ fold change values) in HS blood and HS skin of up-regulated (**a**) and down-regulated (**b**) DETs of blood HS Th_mem_ cells were plotted against each other. Spearman’s correlation coefficient *R*-values are indicated. HS, hidradenitis suppurativa, DETs, differentially expressed transcripts.

Second, we correlated the log_2_ fold change values of the DETs of blood HS Th_mem_ cells versus the log_2_ fold change values of the same transcripts that were calculated for HS skin lesions compared to healthy donor skin. As shown in Figure 4, there was no significant correlation between the changes in blood Th_mem_ cells and in skin for either up-regulated (Figure 4a) or down-regulated (Figure 4b) blood Th_mem_ cell DETs. Overall, these data show a lack of a blood-Th_mem_-cell-specific transcriptomic signature in lesional HS skin and imply that the molecular alterations identified in HS blood Th_mem_ cells are not involved in HS skin pathology. This suggests three possible scenarios for the massive cutaneous presence of T cells, which definitely play a pathogenetic role in the persistence of HS skin lesions: (i) the T cells immigrate into the skin in large numbers before/early in the development of lesions, (ii) they obtain their pathogenic profile only after having immigrated into the skin, or (iii) they arise from the T cells that were already in the skin (e.g., from resident memory cells). The second and third scenario are supported by the presence of the tertiary lymphoid structures in HS lesions [38].

**Figure 5:**
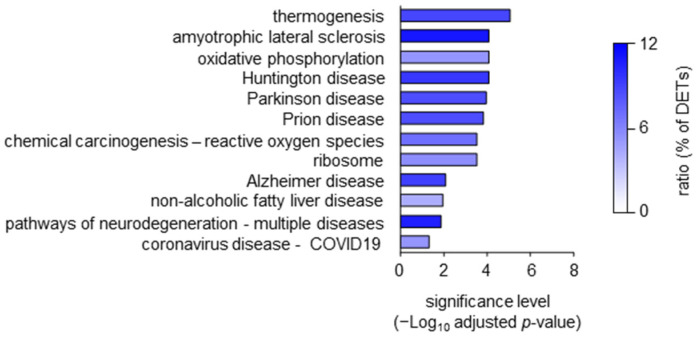
Association of DETs of blood HS Th_mem_ cells with diseases. DETs of blood HS Th_mem_ cells determined as described in Figure 1b were subjected to KEGG pathway enrichment analysis to determine their possible association with human diseases. HS, hidradenitis suppurativa, DETs, differentially expressed transcripts.

Since HS is a complex, systemic disease with multiple comorbidities [1], we wondered whether the DETs detected in blood HS Th_mem_ cells were associated with signatures known from other diseases. As presented in Figure 5, a gene ontology enrichment analysis for human disease association demonstrated a link of the DETs detected in blood HS Th_mem_ cells with different neurological disorders, as well as adipose-tissue-involving thermogenesis, oxidative-stress-mediated carcinogenesis, non-alcoholic fatty liver disease, and virus infection. Interestingly, depression, schizophrenia, and bipolar disorder as well as metabolic alterations and non-alcoholic fatty liver disease were demonstrated to be associated with HS [10,11,17,53,54].

It should be noted that in these analyses, we did not observe any enrichment of transcripts associated with skin disease (Figure 5), supporting our observation described above that in patients with manifest HS skin alterations, changes in the blood Th_mem_ cells are no indicators of the changes in the skin.

**Figure 6:**
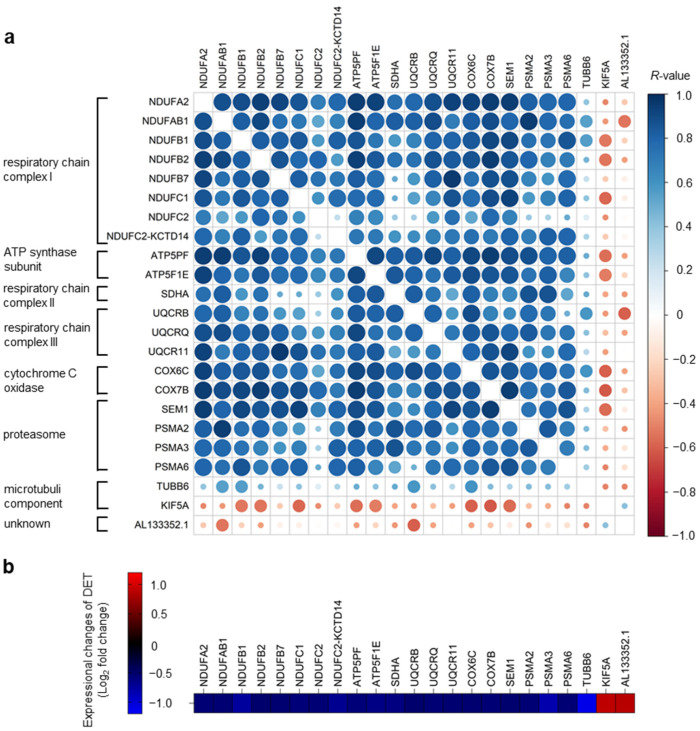
Expression levels of DETs of blood HS Th_mem_ cells associated with different neurological diseases show positive associations with each other. DETs of blood HS Th_mem_ cells were determined as described in Figure 1b and subjected to KEGG pathway enrichment analysis as described in Figure 5. (**a**) Expression levels of DETs of blood HS Th_mem_ cells that were associated with all neurological diseases shown in Figure 5 (amyotrophic lateral sclerosis, Huntington disease, Parkinson disease, Prion disease, Alzheimer disease, Pathways of neurodegeneration—multiple diseases) were correlated to each other. Data are presented as correlation matrix. Spearman’s correlation coefficient *R* and *p*-values are indicated by the color scale (see legend) and size (maximum size: *p* < 0.001, 75%: *p* < 0.01, 50%: *p* < 0.05, 10%: *p* ≥ 0.05) of circles, respectively. (**b**) Log_2_ of fold change expression (Th_mem_ cells isolated from the blood of HS patients versus those from healthy donor participants) values of DETs shown in (**a**) are given using a visual color scale. HS, hidradenitis suppurativa, DETs, differentially expressed transcripts.

Since neurological diseases were the numerically largest group of disorders associated with the DETs of blood HS Th_mem_ cells, we then focused on them. Among the DETs associated with neurological diseases, there was a set of 21 transcripts common to all these disorders (Figure 6a). This comprised: COX6C, SDHA, NDUFB1, UQCRB, NDUFC2-KCTD14, NDUFAB1, UQCR11, NDUFB2, NDUFB7, COX7B, NDUFC2, NDUFA2, NDUFC1, AL133352.1, UQCRQ, KIF5A, SEM1, PSMA6, PSMA2, ATP5PF, ATP5F1E, PSMA3, and TUBB6. These transcripts include elements of the mitochondrial respiratory chain complex 1 (NDUFA2, NDUFAB1, NDUFB1, NDUFB2, NDUFB7, NDUFC1, NDUFC2, NDUFC2-KCTD14), complex 2 (SDHA) and complex 3 (UQCRB, UQCR11, UQCRQ), cytochrome C oxidase (COX6C, COX7B), and ATP-synthase (ATP5PF, ATP5F1E). Furthermore, they included genes coding for the subunits of proteasomes, catalytic protease complexes (SEM1, PSMA2, PSMA3, and PSMA6), and those involved in microtubule composition (TUBB6) and function (KIF5A).

The expression levels of most of these DETs showed a strong positive correlation to each other, suggesting similar regulatory mechanisms of their expression in the blood HS Th_mem_ cells (Figure 6a). Importantly, most of the 21 transcripts common to all the neurological diseases were down-regulated in blood HS Th_mem_ cells compared to the Th_mem_ cells obtained from healthy participants (Figure 6b). Interestingly, a down-regulation of the expression of several ATP-synthase-encoding genes and numerous proteasome subunit genes was recently demonstrated in the brain of patients with schizophrenia [55,56]. More importantly, Song et al. found that the transcripts associated with mitochondrial oxidative phosphorylation have similar expression patterns in blood immune cells and brain tissues in patients with schizophrenia [57]. It should be mentioned that despite distinct clinical symptoms, the accumulation of misfolded or aggregated proteins in the brain is characteristic of many neurodegenerative disorders, such as amyotrophic lateral sclerosis, Huntington’s disease, Parkinson’s disease, or Alzheimer’s disease. Growing evidence suggests that dysregulation in intracellular protein degradation mechanisms (the ubiquitin–proteasome system or autophagy) leads to proteostasis, contributing to the onset and persistence of these diseases [58,59,60,61]. Thus, the enhancement of proteasome-mediated protein degradation is a promising new therapy option for neurodegenerative disorders that is already being investigated [62,63,64]. The identification of the DETs of blood HS Th_mem_ cells associated with neurological diseases raises the hope that some of these molecules might be suitable as blood biomarkers that are predictive for neurological comorbidities in HS. Unfortunately, when recruiting the HS patients in this project, the presence of neurological diseases was not inquired about, so we cannot correlate the expression levels of the identified DETs with the presence or absence of the corresponding neurological disorders. In addition, there was no follow-up in this project; thus, we cannot make any statement as to whether the corresponding HS patients developed neurological diseases. Finally, as the results of the study were not foreseeable when it was planned, patients with only neurological diseases where not recruited as a control group. These are certainly limitations of our research project.

In summary, we identified numerous transcriptomic changes in Th_mem_ cells isolated from the blood of patients with HS. To our knowledge, this is the first publication demonstrating the results of such an analysis in HS patients. The transcriptomic alterations concern proteins involved in nucleotide metabolism and ATP synthesis through oxidation in the mitochondria. The changes identified in HS blood Th_mem_ cells were not found in the HS skin lesions and are unlikely to contribute to the persistence of skin alterations. We consider it a very important finding of this project that the analysis of the blood cells obtained from patients with apparent skin alterations did not provide much insight into the cutaneous HS pathology. Rather, one has the impression that the Th_mem_ cells found in HS patients with developed lesions could be responsible for potential comorbidities. Future studies are required to prove this hypothesis.

## 3. Materials and Methods

### 3.1. Patients and Healthy Donors

Blood samples were obtained from 15 healthy adult volunteers and 15 adult HS patients who did not receive any systemic therapy for at least 3 months before participating in the study. Both participant groups were matched to each other in terms of gender distribution (healthy/HS: 60% female, 40% male), age (healthy: 36.9 ± 2.4; HS: 37.5 ± 3.1) and smoking habit (healthy: 60% smokers, 40% non-smokers; HS: 60% smokers, 40% non-smokers). Skin samples were obtained from healthy participants (n = 4) and patients with HS (n = 3; lesional skin from axillary region).

The collection of all blood and skin samples was approved by the clinical institutional review board of the Charité University Hospital, Berlin, and written informed consent was obtained from all participants. The study was conducted according to the principles of the Declaration of Helsinki.

### 3.2. Cell Isolation

Peripheral blood mononuclear cells (PBMCs) were isolated from the venous blood of patients and healthy donors using density gradient centrifugation as described earlier [65]. CD4^+^ memory T cells were purified from the obtained PBMCs via magnetic cell sorting using the MACSTM system and the memory CD4^+^ T cell isolation kit (negative selection) from Miltenyi Biotec. The purity of the isolated T_mem_ cells, as analyzed by flow cytometry (see below), was 97.63 ± 0.18 (healthy donors) and 96.72 ± 0.52 (HS patients) (Figure S1 and Table S3).

### 3.3. Flow Cytometry

An analysis of the cell purity of the isolated T_mem_ cells was performed via flow cytometry, using the following fluorescent labeled antibodies directed against CD45RA (clone HI10), CD45RO (clone UCHL1), CD3 (clone SK7) (all purchased from BD Biosciences, Heidelberg, Germany), and CD4 (clone 13B8.2) (purchased from Beckmann Coulter, Krefeld, Germany), using a FACS Calibur device (BD Biosciences) as described earlier [65]. Data processing and analysis were performed using associated CellQuest Pro software (version 4.0.2).

### 3.4. Transcriptome Analysis

RNA was isolated from the purified Th_mem_ cells using a Trizol lysing solution (Thermo Fisher, Waltham, MA, USA) according to the manufacturer’s instructions. A library preparation from the total RNA was performed using the TruSeq stranded total RNA library prep kit with ribo-zero gold (Illumina, San Diego, CA, USA) according to the manufacturer’s recommendation, followed by the sequencing of libraries (50 bp single reads; 30 million reads per sample) using a HiSeq 2000 platform (Illumina). The transformation of the fluorescence images into BCL files was accomplished using the Illumina BaseCaller software followed by the demultiplexing of samples to FASTQ files using CASAVA (version 1.8.2). The sequencing quality was checked using FastQC software (www.bioinformatics.babraham.ac.uk/projects/fastqc, accessed on 3 May 2023). Reads were aligned to the hg38 using Salmon, which is available from https://anaconda.org/bioconda/salmon (accessed on 3 May 2023). Quantifications were imported into R and summarized at the gene level. Further processing and analysis steps were carried out using DESeq2, which is available from https://bioconductor.org (accessed on 3 May 2023). Differentially expressed transcripts were determined after the data were fitted to models of negative binomial distributions. Raw *p*-values were fdr-corrected for multiple testing.

Shrinkage of the transcriptome data was performed using the lfcShrink function (type = “normal”) using DESeq2 in R. A heatmap was generated using the inbuilt heatmap function of R with a predefined order of the samples.

The correlation matrix plot was generated using the corrplot package (Wei T, Simko V (2021); R package ‘corrplot’: Visualization of a Correlation Matrix (Version 0.92), available from https://github.com/taiyun/corrplot, accessed on 3 May 2023).

Skin samples from healthy donors and HS patients were processed for transcriptome analysis as previously described [35,38]. The obtained Th_mem_ cell and skin transcriptome data were filtered for protein coding genes, and cutoffs for the differentially expressed transcripts (DETs) were set to an adjusted *p* < 0.01; log_2_ fold change 0.5/−0.5.

### 3.5. Gene Set Enrichment Analysis

An overrepresentation analysis of the differentially expressed genes in terms of gene ontology and in KEGG pathways after DAVID annotation was performed with the clusterProfiler package. The visualization of the DETs of blood HS Th_mem_ cells involved in oxidative phosphorylation and glycolysis was performed with Qiagen IPA software (v01-22-01). For this analysis, the DETs involved in the intracellular processes shown in Figure 2a were used.

### 3.6. Overrepresentation of Regulatory Target Gene Set Analysis

To identify possible master regulators of target genes found to be differentially regulated in the HS Th_mem_ cells, the msigdbr R-package was used (msigdbr: MSigDB Gene Sets for Multiple Organisms in a Tidy Data Format (https://cran.r-project.org/web/packages/msigdbr/msigdbr.pdf, accessed on 3 May 2023); R package version 7.5.1, https://CRAN.R-project.org/package=msigdbr, accessed on 3 May 2023). The enrichment of genes within these sets was determined by the enricher function of the clusterProfiler R-package [66]. Raw *p*-values were fdr-corrected for multiple testing, and an adjusted *p*-value below 0.05 was considered significant.

### 3.7. Further Statistical Analysis

A correlation analysis of the log_2_ fold-change values of the blood Th_mem_ cells versus the HS skin (Spearman’s rank-order correlation test; Figure 4) and a comparison of DET frequencies (*X*_2_ test; Figure 6a) were performed using SPSS software (version 29.0.0.0(241)).

## Figures and Tables

**Table 1:** Demographic and clinical characteristics of the study cohort. HS, hidradenitis suppurativa; n.a., not applicable.

	HS	Healthy
	(n = 15)	(n = 13)
Age, in years; mean ± SEM	37.6 ± 3.1	37.5 ± 2.6
Gender distribution; n (%)		
Females:	9 (60.0)	8 (61.5)
Males:	6 (40.0)	5 (38.5)
Smoking habit; n (%)		
Smokers:	10 (66.6)	7 (53.8)
Son-smokers:	5 (33.3)	6 (46.2)
Disease duration, in years; mean ± SEM	15.2 ± 3.0	n.a.
Positive family history for HS; n (%)	5 (33.3)	n.a.
Hurley score; mean ± SEM	1.4 ± 0.2	n.a.
Sartorius score; mean ± SEM	48.3 ± 11.8	n.a.

**Table 2:** Top 20 up-regulated DETs of Th_mem_ cells from HS patients compared to Th_mem_ cells from healthy participants.

Ensembl ID	Gene Name	Gene Description	Log_2_ Fold Change	*p*
ENSG00000254995	STX16-NPEPL1	syntaxin 16	1.58	5.17 × 10^−7^
ENSG00000123454	DBH	dopamine beta-hydroxylase	1.57	3.88 × 10^−6^
ENSG00000241489	AC244197.3	novel protein	1.54	1.95 × 10^−6^
ENSG00000121454	LHX4	LIM homeobox 4	1.53	6.64 × 10^−6^
ENSG00000009724	MASP2	MBL associated serine protease 2	1.51	3.99 × 10^−6^
ENSG00000151006	PRSS53	serine protease 53	1.49	4.40 × 10^−7^
ENSG00000284946	AC068831.7	novel protein	1.48	7.54 × 10^−6^
ENSG00000167701	GPT	glutamic-pyruvic transaminase	1.48	3.36 × 10^−5^
ENSG00000198838	RYR3	ryanodine receptor 3	1.47	1.52 × 10^−4^
ENSG00000255423	EBLN2	endogenous Bornavirus-like nucleoprotein 2	1.45	1.10 × 10^−5^
ENSG00000146373	RNF217	ring finger protein 217	1.44	3.20 × 10^−5^
ENSG00000187726	DNAJB13	DnaJ homolog subfamily B member 13	1.43	8.80 × 10^−7^
ENSG00000225987	PBX2	PBX homeobox 2	1.38	2.17 × 10^−5^
ENSG00000284981	AC093668.2	novel protein	1.38	4.10 × 10^−7^
ENSG00000138834	MAPK8IP3	mitogen-activated protein kinase 8 interacting protein 3	1.38	4.69 × 10^−12^
ENSG00000239732	TLR9	Toll-like receptor 9	1.35	2.54 × 10^−4^
ENSG00000111886	GABRR2	gamma-aminobutyric acid type A receptor subunit rho2	1.35	6.89 × 10^−4^
ENSG00000130827	PLXNA3	plexin A3	1.35	1.07 × 10^−7^
ENSG00000144802	NFKBIZ	NFKB inhibitor zeta	1.33	6.00 × 10^−8^
ENSG00000283199	C13orf46	uncharacterized protein	1.32	2.45 × 10^−5^

**Table 3:** Top 20 down-regulated DETs of Th_mem_ cells from HS patients compared to Th_mem_ cells from healthy participants.

Ensembl ID	Gene Name	Gene Description	Log_2_ Fold Change	*p*
ENSG00000140675	SLC5A2	solute carrier family 5 member 2	−1.61	7.66 × 10^−6^
ENSG00000188452	CERKL	ceramide kinase-like	−1.55	5.38 × 10^−5^
ENSG00000104213	PDGFRL	Platelet-derived growth factor receptor-like	−1.29	4.36 × 10^−4^
ENSG00000163806	SPDYA	speedy/RINGO cell cycle regulator family member A	−1.28	1.01 × 10^−3^
ENSG00000257529	RPL36A-HNRNPH2	RPL36A-HNRNPH2 readthrough	−1.25	2.84 × 10^−5^
ENSG00000156050	FAM161B	family with sequence similarity 161, member B	−1.24	1.19 × 10^−3^
ENSG00000096150	RPS18	ribosomal protein S18	−1.22	6.93 × 10^−5^
ENSG00000164142	FAM160A1	FHF complex subunit HOOK interacting protein 1A	−1.22	1.22 × 10^−3^
ENSG00000168350	DEGS2	delta 4-desaturase	−1.21	2.69 × 10^−3^
ENSG00000168000	BSCL2	BSCL2 lipid droplet biogenesis associated, seipin	−1.21	4.43 × 10^−4^
ENSG00000162613	FUBP1	far upstream element binding protein 1	−1.18	8.48 × 10^−5^
ENSG00000253304	TMEM200B	transmembrane protein 200B	−1.18	5.61 × 10^−4^
ENSG00000108556	CHRNE	cholinergic receptor nicotinic epsilon subunit	−1.17	1.31 × 10^−3^
ENSG00000137731	FXYD2	FXYD domain containing ion transport regulator 2	−1.14	2.11 × 10^−5^
ENSG00000227507	LTB	lymphotoxin beta	−1.13	6.89 × 10^−4^
ENSG00000255508	AP002990.1	uncharacterized protein	−1.12	1.09 × 10^−3^
ENSG00000267179	AC008770.2	uncharacterized protein	−1.11	6.45 × 10^−3^
ENSG00000176014	TUBB6	tubulin beta 6 class V	−1.10	6.82 × 10^−3^
ENSG00000167565	SERTAD3	SERTA domain containing 3	−1.09	2.76 × 10^−4^
ENSG00000226492	CUTA	acetylcholinesterase-associated protein	−1.08	3.55 × 10^−4^

## Data Availability

The main data are presented within the figures of the manuscript. Further study material will be made available upon request to the corresponding authors to the extent permissible by law.

## Supplementary Materials

The following supporting information can be downloaded at: https://www.mdpi.com/article/10.3390/ijms24108854/s1.

## References

[1] Sabat R., Jemec G.B.E., Matusiak L., Kimball A.B., Prens E., Wolk K. (2020). Hidradenitis suppurativa. Nat. Rev. Dis. Primers.

[2] Prens L.M., Bouwman K., Troelstra L.D., Prens E.P., Alizadeh B.Z., Horvath B. (2022). New insights in hidradenitis suppurativa from a population-based Dutch cohort: Prevalence, smoking behaviour, socioeconomic status and comorbidities. Br. J. Dermatol..

[3] Sabat R., Tsaousi A., Ghoreschi K., Wolk K., Schneider-Burrus S. (2022). Sex-disaggregated population analysis in patients with hidradenitis suppurativa. Front. Med..

[4] Fabbrocini G., Ruina G., Giovanardi G., Dini V., Raone B., Venturini M., Caro R.D.C., Veraldi S., Cannavò S.P., Merlo G. (2022). Hidradenitis Suppurativa in a Large Cohort of Italian Patients: Evaluation of the Burden of Disease. Dermatology.

[5] Patel A., Patel A., Solanki D., Mansuri U., Singh A., Sharma P., Solanki S. (2022). Hidradenitis Suppurativa in the United States: Insights From the National Inpatient Sample (2008–2017) on Contemporary Trends in Demographics, Hospitalization Rates, Chronic Comorbid Conditions, and Mortality. Cureus.

[6] Schneider-Burrus S., Tsaousi A., Barbus S., Huss-Marp J., Witte K., Wolk K., Fritz B., Sabat R. (2021). Features Associated with Quality of Life Impairment in Hidradenitis Suppurativa Patients. Front. Med..

[7] Montero-Vilchez T., Diaz-Calvillo P., Rodriguez-Pozo J.A., Cuenca-Barrales C., Martinez-Lopez A., Arias-Santiago S., Molina-Leyva A. (2021). The Burden of Hidradenitis Suppurativa Signs and Symptoms in Quality of Life: Systematic Review and Meta-Analysis. Int. J. Environ. Res. Public Health.

[8] Kurek A., Peters E.M., Chanwangpong A., Sabat R., Sterry W., Schneider-Burrus S. (2012). Profound disturbances of sexual health in patients with acne inversa. J. Am. Acad. Dermatol..

[9] Singh R., Kelly K.A., Senthilnathan A., Feldman S.R., Pichardo R.O. (2022). Stigmatization, a social perception which may have a debilitating impact on hidradenitis suppurativa patients: An observational study. Arch. Dermatol. Res..

[10] Sabat R., Chanwangpong A., Schneider-Burrus S., Metternich D., Kokolakis G., Kurek A., Philipp S., Uribe D., Wolk K., Sterry W. (2012). Increased prevalence of metabolic syndrome in patients with acne inversa. PLoS ONE.

[11] Gau S.Y., Hsiao Y.P., Liao W.C., Ma K.S., Wu M.C. (2022). Risk of liver dysfunction and non-alcoholic fatty liver diseases in people with hidradenitis suppurativa: A systematic review and meta-analysis of real-world evidences. Front. Immunol..

[12] Gonzalez-Lopez M.A., Hernandez J.L., Lacalle M., Mata C., Lopez-Escobar M., Lopez-Mejias R., Portilla V., Fuentevilla P., Corrales A., González-Vela M.C. (2016). Increased prevalence of subclinical atherosclerosis in patients with hidradenitis suppurativa (HS). J. Am. Acad. Dermatol..

[13] Reddy S., Strunk A., Jemec G.B.E., Garg A. (2020). Incidence of Myocardial Infarction and Cerebrovascular Accident in Patients with Hidradenitis Suppurativa. JAMA Dermatol..

[14] Schneider-Burrus S., Witte-Haendel E., Christou D., Rigoni B., Sabat R., Diederichs G. (2016). High Prevalence of Back Pain and Axial Spondyloarthropathy in Patients with Hidradenitis Suppurativa. Dermatology.

[15] Almuhanna N., Finstrad A., Alhusayen R. (2021). Association between Hidradenitis Suppurativa and Inflammatory Arthritis: A Systematic Review and Meta-Analysis. Dermatology.

[16] Schneeweiss M.C., Kirchgesner J., Wyss R., Jin Y., York C., Merola J.F., Mostaghimi A., Silverberg J.I., Schneeweiss S., Glynn R.J. (2022). Occurrence of inflammatory bowel disease in patients with chronic inflammatory skin diseases: A cohort study: Classification: Epidemiology. Br. J. Dermatol..

[17] Huilaja L., Tiri H., Jokelainen J., Timonen M., Tasanen K. (2018). Patients with Hidradenitis Suppurativa Have a High Psychiatric Disease Burden: A Finnish Nationwide Registry Study. J. Investig. Dermatol..

[18] Phan K., Huo Y.R., Smith S.D. (2020). Hidradenitis suppurativa and psychiatric comorbidities, suicides and substance abuse: Systematic review and meta-analysis. Ann. Transl. Med..

[19] Schneider-Burrus S., Kalus S., Fritz B., Wolk K., Gomis-Kleindienst S., Sabat R. (2023). The impact of hidradenitis suppurativa on professional life. Br. J. Dermatol..

[20] Scholl L., Schneider-Burrus S., Fritz B., Sabat R., Bechara F.G. (2023). The impact of surgical interventions on the psychosocial well-being of patients with hidradenitis suppurativa. J. Dtsch. Dermatol. Ges..

[21] Ocker L., Abu Rached N., Seifert C., Scheel C., Bechara F.G. (2022). Current Medical and Surgical Treatment of Hidradenitis Suppurativa-A Comprehensive Review. J. Clin. Med..

[22] Witte K., Sabat R., Witte-Handel E., Ghoreschi K., Wolk K. (2022). Phytotherapeuthics Affecting the IL-1/IL-17/G-CSF Axis: A Complementary Treatment Option for Hidradenitis Suppurativa?. Int. J. Mol. Sci..

[23] Gamell C., Bankovacki A., Scalzo-Inguanti K., Sedgmen B., Alhamdoosh M., Gail E., Turkovic L., Millar C., Johnson L., Wahlsten M. (2023). CSL324, a G-CSF receptor antagonist, blocks neutrophil migration markers that are upregulated in hidradenitis suppurativa. Br. J. Dermatol..

[24] Swierczewska Z., Lewandowski M., Surowiecka A., Baranska-Rybak W. (2022). Immunomodulatory Drugs in the Treatment of Hidradenitis Suppurativa-Possibilities and Limitations. Int. J. Mol. Sci..

[25] Shih T., De D., Daveluy S.D., Hogeling M., Lowes M.A., Sayed C., Shi V.Y., Hsiao J.L. (2022). Real-World Considerations of Candidacy for Biologics in Hidradenitis Suppurativa. Am. J. Clin. Dermatol..

[26] Dos Santos-Silva C.A., Tricarico P.M., Vilela L.M.B., Roldan-Filho R.S., Amador V.C., d’Adamo A.P., Rêgo M.d.S., Benko-Iseppon A.M., Crovella S. (2021). Plant Antimicrobial Peptides as Potential Tool for Topic Treatment of Hidradenitis Suppurativa. Front. Microbiol..

[27] Wolk K., Join-Lambert O., Sabat R. (2020). Aetiology and pathogenesis of hidradenitis suppurativa. Br. J. Dermatol..

[28] Abu Rached N., Gambichler T., Dietrich J.W., Ocker L., Seifert C., Stockfleth E., Bechara F.G. (2022). The Role of Hormones in Hidradenitis Suppurativa: A Systematic Review. Int. J. Mol. Sci..

[29] Wolk K., Warszawska K., Hoeflich C., Witte E., Schneider-Burrus S., Witte K., Kunz S., Buss A., Roewert H.J., Krause M. (2011). Deficiency of IL-22 contributes to a chronic inflammatory disease: Pathogenetic mechanisms in acne inversa. J. Immunol..

[30] Swierczewska Z., Lewandowski M., Surowiecka A., Baranska-Rybak W. (2022). Microbiome in Hidradenitis Suppurativa—What We Know and Where We Are Heading. Int. J. Mol. Sci..

[31] Wolk K., Wenzel J., Tsaousi A., Witte-Handel E., Babel N., Zelenak C., Volk H.-D., Sterry W., Schneider-Burrus S., Sabat R. (2017). Lipocalin-2 is expressed by activated granulocytes and keratinocytes in affected skin and reflects disease activity in acne inversa/hidradenitis suppurativa. Br. J. Dermatol..

[32] Di Caprio R., Balato A., Caiazzo G., Lembo S., Raimondo A., Fabbrocini G., Monfrecola G. (2017). IL-36 cytokines are increased in acne and hidradenitis suppurativa. Arch. Dermatol. Res..

[33] Thomi R., Kakeda M., Yawalkar N., Schlapbach C., Hunger R.E. (2017). Increased expression of the interleukin-36 cytokines in lesions of hidradenitis suppurativa. J. Eur. Acad. Dermatol. Venereol..

[34] Hessam S., Sand M., Gambichler T., Skrygan M., Ruddel I., Bechara F.G. (2018). Interleukin-36 in hidradenitis suppurativa: Evidence for a distinctive proinflammatory role and a key factor in the development of an inflammatory loop. Br. J. Dermatol..

[35] Witte-Handel E., Wolk K., Tsaousi A., Irmer M.L., Mossner R., Shomroni O., Lingner T., Witte K., Kunkel D., Salinas G. (2019). The IL-1 Pathway Is Hyperactive in Hidradenitis Suppurativa and Contributes to Skin Infiltration and Destruction. J. Investig. Dermatol..

[36] Scala E., Di Caprio R., Cacciapuoti S., Caiazzo G., Fusco A., Tortorella E., Fabbrocini G., Balato A. (2019). A new T helper 17 cytokine in hidradenitis suppurativa: Antimicrobial and proinflammatory role of interleukin-26. Br. J. Dermatol..

[37] Wolk K., Brembach T.C., Simaite D., Bartnik E., Cucinotta S., Pokrywka A., Irmer M., Triebus J., Witte-Händel E., Salinas G. (2021). Activity and components of the granulocyte colony-stimulating factor pathway in hidradenitis suppurativa. Br. J. Dermatol..

[38] Sabat R., Simaite D., Gudjonsson J.E., Brembach T.C., Witte K., Krause T., Kokolakis G., Bartnik E., Nikolaou C., Rill N. (2022). Neutrophilic granulocyte-derived B-cell activating factor supports B cells in skin lesions in hidradenitis suppurativa. J. Allergy Clin. Immunol..

[39] Del Duca E., Morelli P., Bennardo L., Di Raimondo C., Nistico S.P. (2020). Cytokine Pathways and Investigational Target Therapies in Hidradenitis Suppurativa. Int. J. Mol. Sci..

[40] Glatt S., Jemec G.B.E., Forman S., Sayed C., Schmieder G., Weisman J., Rolleri R., Seegobin S., Baeten D., Ionescu L. (2021). Efficacy and Safety of Bimekizumab in Moderate to Severe Hidradenitis Suppurativa: A Phase 2, Double-blind, Placebo-Controlled Randomized Clinical Trial. JAMA Dermatol..

[41] Kimball A.B., Loesche C., Prens E.P., Bechara F.G., Weisman J., Rozenberg I., Jarvis P., Peters T., Roth L., Wieczorek G. (2022). IL-17A is a pertinent therapeutic target for moderate-to-severe hidradenitis suppurativa: Combined results from a pre-clinical and phase II proof-of-concept study. Exp. Dermatol..

[42] Sabat R., Wolk K., Loyal L., Docke W.D., Ghoreschi K. (2019). T cell pathology in skin inflammation. Semin. Immunopathol..

[43] Schnell A., Littman D.R., Kuchroo V.K. (2023). T(H)17 cell heterogeneity and its role in tissue inflammation. Nat. Immunol..

[44] Moran B., Sweeney C.M., Hughes R., Malara A., Kirthi S., Tobin A.M., Kirby B., Fletcher J.M. (2017). Hidradenitis Suppurativa Is Characterized by Dysregulation of the Th17:Treg Cell Axis, Which Is Corrected by Anti-TNF Therapy. J. Investig. Dermatol..

[45] Remedios K.A., Zirak B., Sandoval P.M., Lowe M.M., Boda D., Henley E., Bhattrai S., Scharschmidt T.C., Liao W., Naik H.B. (2018). The TNFRSF members CD27 and OX40 coordinately limit T(H)17 differentiation in regulatory T cells. Sci. Immunol..

[46] Hessam S., Gambichler T., Hoxtermann S., Skrygan M., Sand M., Garcovich S., Meyer T., Stockfleth E., Bechara F.G. (2020). Frequency of circulating subpopulations of T-regulatory cells in patients with hidradenitis suppurativa. J. Eur. Acad. Dermatol. Venereol..

[47] Kono M. (2022). New insights into the metabolism of Th17 cells. Immunol. Med..

[48] Spiljar M., Kuchroo V.K. (2022). Metabolic regulation and function of T helper cells in neuroinflammation. Semin. Immunopathol..

[49] Chakraborty S., Khamaru P., Bhattacharyya A. (2022). Regulation of immune cell metabolism in health and disease: Special focus on T and B cell subsets. Cell Biol. Int..

[50] Peng Q., Zhou M., Zuo S., Liu Y., Li X., Yang Y., He Q., Yu X., Zhou J., He Z. (2021). Nuclear Factor Related to KappaB Binding Protein (NFRKB) Is a Telomere-Associated Protein and Involved in Liver Cancer Development. DNA Cell Biol..

[51] Wang S., Bleeck A., Nadif Kasri N., Kleefstra T., van Rhijn J.R., Schubert D. (2021). SETD1A Mediated H3K4 Methylation and Its Role in Neurodevelopmental and Neuropsychiatric Disorders. Front. Mol. Neurosci..

[52] Nakamura T., Takata A. (2023). The molecular pathology of schizophrenia: An overview of existing knowledge and new directions for future research. Mol. Psychiatry.

[53] Kurek A., Johanne Peters E.M., Sabat R., Sterry W., Schneider-Burrus S. (2013). Depression is a frequent co-morbidity in patients with acne inversa. J. Dtsch. Dermatol. Ges..

[54] Caccavale S., Tancredi V., Boccellino M.P., Babino G., Fulgione E., Argenziano G. (2023). Hidradenitis Suppurativa Burdens on Mental Health: A Literature Review of Associated Psychiatric Disorders and Their Pathogenesis. Life.

[55] Hertzberg L., Maggio N., Muler I., Yitzhaky A., Majer M., Haroutunian V., Zuk O., Katsel P., Domany E., Weiser M. (2021). Comprehensive Gene Expression Analysis Detects Global Reduction of Proteasome Subunits in Schizophrenia. Schizophr. Bull..

[56] Katz Shroitman N., Yitzhaky A., Ben Shachar D., Gurwitz D., Hertzberg L. (2023). Meta-analysis of brain samples of individuals with schizophrenia detects down-regulation of multiple ATP synthase encoding genes in both females and males. J. Psychiatr. Res..

[57] Song X., Liu Y., Pu J., Gui S., Zhong X., Chen X., Chen X., Wang H., Cheng K., Zhao L. (2021). Transcriptomics Analysis Reveals Shared Pathways in Peripheral Blood Mononuclear Cells and Brain Tissues of Patients with Schizophrenia. Front. Psychiatry.

[58] Amrutha K., Mishra A., Singh S. (2022). Implications of intracellular protein degradation pathways in Parkinson’s disease and therapeutics. J. Neurosci. Res..

[59] Tecalco-Cruz A.C., Pedraza-Chaverri J., Briones-Herrera A., Cruz-Ramos E., Lopez-Canovas L., Zepeda-Cervantes J. (2022). Protein degradation-associated mechanisms that are affected in Alzheimer s disease. Mol. Cell. Biochem..

[60] Hommen F., Bilican S., Vilchez D. (2022). Protein clearance strategies for disease intervention. J. Neural Transm..

[61] Dilliott A.A., Andary C.M., Stoltz M., Petropavlovskiy A.A., Farhan S.M.K., Duennwald M.L. (2022). DnaJC7 in Amyotrophic Lateral Sclerosis. Int. J. Mol. Sci..

[62] Benn J.A., Mukadam A.S., McEwan W.A. (2022). Targeted protein degradation using intracellular antibodies and its application to neurodegenerative disease. Semin. Cell Dev. Biol..

[63] Sasso J.M., Tenchov R., Wang D., Johnson L.S., Wang X., Zhou Q.A. (2022). Molecular Glues: The Adhesive Connecting Targeted Protein Degradation to the Clinic. Biochemistry.

[64] George D.E., Tepe J.J. (2021). Advances in Proteasome Enhancement by Small Molecules. Biomolecules.

[65] Wolk K., Mitsui H., Witte K., Gellrich S., Gulati N., Humme D., Witte E., Gonsior M., Beyer M., Kadin M.E. (2014). Deficient cutaneous antibacterial competence in cutaneous T-cell lymphomas: Role of Th2-mediated biased Th17 function. Clin. Cancer Res..

[66] Wu T., Hu E., Xu S., Chen M., Guo P., Dai Z., Feng T., Zhou L., Tang W., Zhan L. (2021). clusterProfiler 4.0: A universal enrichment tool for interpreting omics data. Innovation.

