# Supplementary material for: Blood T Helper Memory Cells: A Tool for Studying Skin Inflammation in HS?
Source: Int J Mol Sci. 2023 May 16;24(10):8854. doi: 10.3390/ijms24108854 (PMC10219182; doi:10.3390/ijms24108854)
Supplement: Supplementary file 1 [file ijms-24-08854-s001.zip › ijms-2315160-supplementary.pdf]

SUPPLEMENTARY MATERIAL

Blood T Helper Memory Cells: A Tool for Studying Skin Inflammation in HS?

Katrin Witte [1, 2, 3], Sylke Schneider-Burrus [1, 4], Gabriela Salinas [5], Rotraut Mössner [6], Kamran Ghoreschi [7], Kerstin Wolk [1, 2, 3], Robert Sabat [1, 2]

[1] Psoriasis Research and Treatment Center, Charité — Universitätsmedizin Berlin, Corporate Member of Freie Universität Berlin and Humboldt-Universität zu Berlin, 10117 Berlin, Germany

[2] Interdisciplinary Group of Molecular Immunopathology, Dermatology/Medical Immunology, Charité — Universitätsmedizin Berlin, Corporate Member of Freie Universität Berlin and Humboldt-Universität zu Berlin, 10117 Berlin, Germany

[3] Berlin Institute of Health Center for Regenerative Therapies (BCRT), Charité — Universitätsmedizin Berlin, Corporate Member of Freie Universität Berlin and Humboldt-Universität zu Berlin, 13353 Berlin, Germany

[4] Center for Dermatosurgery, Havelklinik Berlin, 13595 Berlin, Germany

[5] NGS-Integrative Genomics Core Unit, Institute of Human Genetics, University Medical Center Göttingen, 37073 Göttingen, Germany

[6] Department of Dermatology, Georg-August-University Goettingen, 37073 Goettingen, Germany

[7] Department of Dermatology, Venereology and Allergology, Charité—Universitätsmedizin Berlin, Corporate Member of Freie Universität Berlin and Humboldt-Universität zu Berlin, 10117 Berlin, Germany

Correspondence:

robert.sabat@charite.de; Tel.: +49-03-450-518625; Fax: +49-03-450-518964

kerstin.wolk@charite.de; Tel.: +49-03-450-518009; Fax: +49-03-450-518964

Running title:

Blood $CD4^+$ T cells in HS

Supplementary Figures

Figure S1. Analysis of the purity of isolated blood Th$_{mem}$. cells. Th$_{mem}$ cells were separated from venous blood of patients with HS and healthy participants by density gradient centrifugation and magnetic cell sorting as described in the Materials and methods section. The purity of separated Th$_{mem}$ cells was then assessed by flow cytometry as also described in the Materials and methods section. Representative dot plots of Th$_{mem}$ cells separated from two healthy donors (a) and two HS patients (b) are demonstrated.

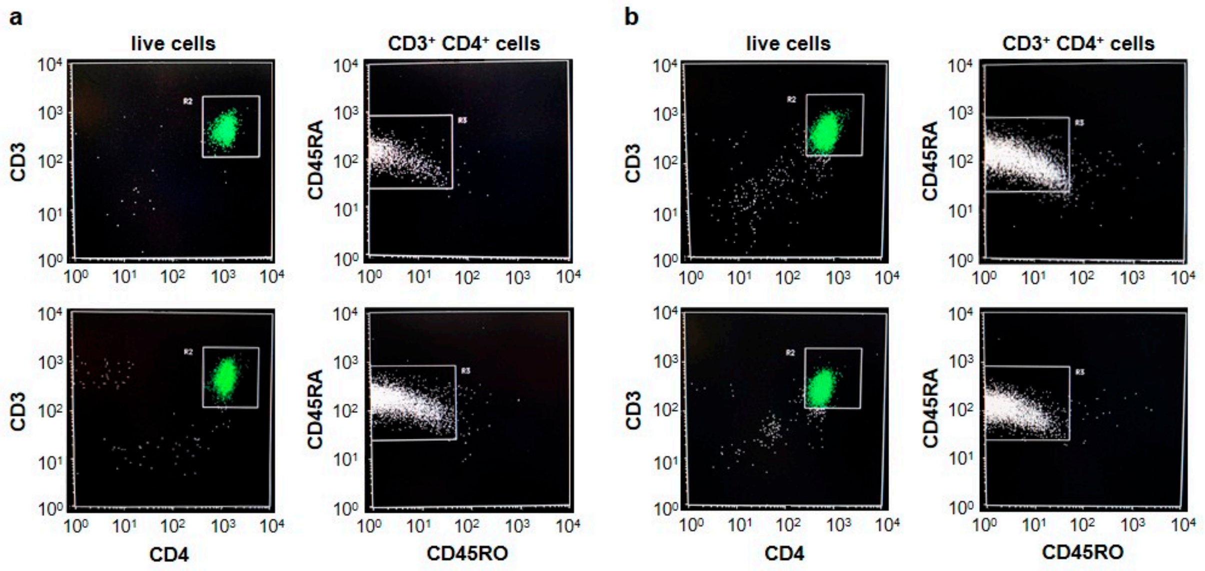

Supplementary Tables

Table S1. List of DETs, regulated in Th_{mem} cells from HS patients compared to Th_{mem} cells from healthy participants (cutoff: adjusted *P*<0.01; log_2 fold change 0.5/-0.5). HS, hidradenitis suppurativa.

Ensembl ID	Gene name	log_2FoldChange	adjusted *P* -value	Gene_biotype
ENSG00000047578	**KIAA0556**	0.983	1.29E-12	protein_coding
ENSG00000138834	**MAPK8IP3**	1.377	4.69E-12	protein_coding
ENSG00000077044	**DGKD**	0.805	3.55E-11	protein_coding
ENSG00000134884	**ARGLU1**	0.933	1.22E-10	protein_coding
ENSG00000124181	**PLCG1**	0.963	2.43E-10	protein_coding
ENSG00000160613	**PCSK7**	0.510	5.17E-10	protein_coding
ENSG00000161217	**PCYT1A**	0.725	1.31E-09	protein_coding
ENSG00000185163	**DDX51**	0.550	1.60E-09	protein_coding
ENSG00000099904	**ZDHHC8**	0.856	2.29E-09	protein_coding
ENSG00000188566	**NDOR1**	0.776	8.74E-09	protein_coding
ENSG00000065029	**ZNF76**	0.518	1.93E-08	protein_coding
ENSG00000005206	**SPPL2B**	0.912	4.44E-08	protein_coding
ENSG00000144802	**NFKBIZ**	1.333	6.00E-08	protein_coding
ENSG00000282725	**CORO7**	0.604	6.00E-08	protein_coding
ENSG00000176953	**NFATC2IP**	0.660	6.24E-08	protein_coding
ENSG00000204264	**PSMB8**	0.762	7.17E-08	protein_coding
ENSG00000102349	**KLF8**	0.963	1.07E-07	protein_coding
ENSG00000130827	**PLXNA3**	1.346	1.07E-07	protein_coding
ENSG00000131584	**ACAP3**	0.988	1.09E-07	protein_coding
ENSG00000243696	**AC006254.1**	1.276	1.99E-07	protein_coding
ENSG00000140688	**C16orf58**	0.520	2.59E-07	protein_coding
ENSG00000141503	**MINK1**	0.945	3.81E-07	protein_coding
ENSG00000284981	**AC093668.2**	1.384	4.10E-07	protein_coding
ENSG00000151006	**PRSS53**	1.491	4.40E-07	protein_coding
ENSG00000139625	**MAP3K12**	0.693	4.75E-07	protein_coding
ENSG00000131697	**NPHP4**	0.751	4.91E-07	protein_coding
ENSG00000254995	**STX16-NPEPL1**	1.578	5.17E-07	protein_coding
ENSG00000125447	**GGA3**	0.652	5.48E-07	protein_coding
ENSG00000126012	**KDM5C**	0.831	6.16E-07	protein_coding
ENSG00000198218	**QRICH1**	0.567	6.57E-07	protein_coding
ENSG00000124222	**STX16**	0.766	8.05E-07	protein_coding
ENSG00000187726	**DNAJB13**	1.433	8.80E-07	protein_coding
ENSG00000184381	**PLA2G6**	0.808	9.15E-07	protein_coding
ENSG00000278372	**MYO19**	0.780	1.01E-06	protein_coding
ENSG00000005844	**ITGAL**	0.615	1.15E-06	protein_coding
ENSG00000108384	**RAD51C**	-0.622	1.24E-06	protein_coding
ENSG00000130940	**CASZ1**	1.226	1.32E-06	protein_coding
ENSG00000178971	**CTC1**	0.946	1.58E-06	protein_coding
ENSG00000285171	**AL590764.2**	0.967	1.75E-06	protein_coding
ENSG00000178038	**ALS2CL**	0.720	1.78E-06	protein_coding
ENSG00000241489	**AC244197.3**	1.541	1.95E-06	protein_coding
ENSG00000176396	**EID2**	-0.586	2.28E-06	protein_coding
ENSG00000268350	**FAM156A**	1.035	3.01E-06	protein_coding
ENSG00000254536	**AL360181.3**	1.025	3.42E-06	protein_coding
ENSG00000024862	**CCDC28A**	-0.634	3.46E-06	protein_coding
ENSG00000119878	**CRIPT**	-0.701	3.57E-06	protein_coding
ENSG00000114745	**GORASP1**	0.657	3.69E-06	protein_coding
ENSG00000123454	**DBH**	1.567	3.88E-06	protein_coding
ENSG00000009724	**MASP2**	1.511	3.99E-06	protein_coding

ENSG00000103091	**WDR59**	0.629	3.99E-06	protein_coding
ENSG00000111752	**PHC1**	0.546	4.15E-06	protein_coding
ENSG00000067048	**DDX3Y**	0.839	4.16E-06	protein_coding
ENSG00000108799	**EZH1**	0.586	4.16E-06	protein_coding
ENSG00000170382	**LRRN2**	1.235	4.63E-06	protein_coding
ENSG00000130475	**FCHO1**	0.536	4.97E-06	protein_coding
ENSG00000168970	**JMJD7-PLA2G4B**	1.212	4.99E-06	protein_coding
ENSG00000185189	**NRBP2**	0.885	5.28E-06	protein_coding
ENSG00000167524	**RSKR**	1.110	5.90E-06	protein_coding
ENSG00000121454	**LHX4**	1.526	6.64E-06	protein_coding
ENSG00000205138	**SDHAF1**	-0.771	6.83E-06	protein_coding
ENSG00000163660	**CCNL1**	0.725	6.97E-06	protein_coding
ENSG00000183682	**BMP8A**	1.187	7.07E-06	protein_coding
ENSG00000124207	**CSE1L**	-0.526	7.09E-06	protein_coding
ENSG00000174173	**TRMT10C**	-0.541	7.09E-06	protein_coding
ENSG00000162526	**TSSK3**	1.138	7.53E-06	protein_coding
ENSG00000284946	**AC068831.7**	1.477	7.54E-06	protein_coding
ENSG00000140675	**SLC5A2**	-1.609	7.66E-06	protein_coding
ENSG00000100567	**PSMA3**	-0.808	9.11E-06	protein_coding
ENSG00000141959	**PFKL**	0.943	9.11E-06	protein_coding
ENSG00000109534	**GAR1**	-0.549	9.54E-06	protein_coding
ENSG00000122515	**ZMIZ2**	0.752	9.61E-06	protein_coding
ENSG00000178209	**PLEC**	0.764	9.85E-06	protein_coding
ENSG00000189007	**ADAT2**	0.903	9.85E-06	protein_coding
ENSG00000205903	**ZNF316**	0.771	9.87E-06	protein_coding
ENSG00000163013	**FBXO41**	0.650	1.04E-05	protein_coding
ENSG00000147251	**DOCK11**	-0.688	1.05E-05	protein_coding
ENSG00000255423	**EBLN2**	1.453	1.10E-05	protein_coding
ENSG00000095564	**BTAF1**	1.015	1.16E-05	protein_coding
ENSG00000011243	**AKAP8L**	0.553	1.35E-05	protein_coding
ENSG00000162004	**CCDC78**	1.038	1.80E-05	protein_coding
ENSG00000169045	**HNRNPH1**	0.642	1.80E-05	protein_coding
ENSG00000091592	**NLRP1**	0.778	1.85E-05	protein_coding
ENSG00000184677	**ZBTB40**	0.641	2.05E-05	protein_coding
ENSG00000110911	**SLC11A2**	0.549	2.06E-05	protein_coding
ENSG00000167280	**ENGASE**	0.875	2.08E-05	protein_coding
ENSG00000137731	**FXYD2**	-1.135	2.11E-05	protein_coding
ENSG00000142687	**KIAA0319L**	0.707	2.11E-05	protein_coding
ENSG00000175787	**ZNF169**	0.934	2.11E-05	protein_coding
ENSG00000186815	**TPCN1**	0.695	2.11E-05	protein_coding
ENSG00000037042	**TUBG2**	0.602	2.16E-05	protein_coding
ENSG00000225987	**PBX2**	1.384	2.17E-05	protein_coding
ENSG00000259330	**INAFM2**	-0.716	2.20E-05	protein_coding
ENSG00000283199	**C13orf46**	1.319	2.45E-05	protein_coding
ENSG00000161202	**DVL3**	0.527	2.55E-05	protein_coding
ENSG00000168066	**SF1**	0.512	2.71E-05	protein_coding
ENSG00000126705	**AHDC1**	0.726	2.76E-05	protein_coding
ENSG00000257529	**RPL36A-HNRNPH**	-1.245	2.84E-05	protein_coding
ENSG00000127511	**SIN3B**	0.796	2.89E-05	protein_coding
ENSG00000166130	**IKBIP**	-0.733	3.18E-05	protein_coding

ENSG00000146373	**RNF217**	1.443	3.20E-05	protein_coding
ENSG00000167701	**GPT**	1.475	3.36E-05	protein_coding
ENSG00000140545	**MFGE8**	0.563	3.39E-05	protein_coding
ENSG00000172216	**CEBPB**	-0.656	3.72E-05	protein_coding
ENSG00000167615	**LENG8**	1.177	3.84E-05	protein_coding
ENSG00000213190	**MLLT11**	0.586	3.84E-05	protein_coding
ENSG00000284431	**AL022238.4**	1.297	4.22E-05	protein_coding
ENSG00000111450	**STX2**	0.516	4.32E-05	protein_coding
ENSG00000131779	**PEX11B**	-0.575	4.44E-05	protein_coding
ENSG00000167785	**ZNF558**	0.802	4.61E-05	protein_coding
ENSG00000197948	**FCHSD1**	0.777	4.64E-05	protein_coding
ENSG00000139908	**TSSK4**	1.206	4.76E-05	protein_coding
ENSG00000168303	**MPLKIP**	-0.521	5.07E-05	protein_coding
ENSG00000163536	**SERPINI1**	-0.694	5.08E-05	protein_coding
ENSG00000160948	**VPS28**	1.174	5.11E-05	protein_coding
ENSG00000066855	**MTFR1**	0.896	5.31E-05	protein_coding
ENSG00000074855	**ANO8**	0.805	5.32E-05	protein_coding
ENSG00000188452	**CERKL**	-1.548	5.38E-05	protein_coding
ENSG00000101343	**CRNKL1**	-0.667	5.48E-05	protein_coding
ENSG00000164828	**SUN1**	0.525	5.48E-05	protein_coding
ENSG00000261408	**TEN1-CDK3**	1.134	5.48E-05	protein_coding
ENSG00000137547	**MRPL15**	-0.542	5.65E-05	protein_coding
ENSG00000167302	**TEPSIN**	0.619	5.88E-05	protein_coding
ENSG00000169612	**RAMAC**	-0.500	5.88E-05	protein_coding
ENSG00000256060	**TRAPPC2B**	-0.713	5.90E-05	protein_coding
ENSG00000146083	**RNF44**	0.600	5.95E-05	protein_coding
ENSG00000198690	**FAN1**	0.924	5.95E-05	protein_coding
ENSG00000197217	**ENTPD4**	0.631	5.96E-05	protein_coding
ENSG00000139631	**CSAD**	1.027	6.10E-05	protein_coding
ENSG00000168781	**PPIP5K1**	0.951	6.17E-05	protein_coding
ENSG00000132361	**CLUH**	0.597	6.27E-05	protein_coding
ENSG00000143256	**PFDN2**	-0.609	6.27E-05	protein_coding
ENSG00000163166	**IWS1**	-0.565	6.27E-05	protein_coding
ENSG00000103168	**TAF1C**	0.863	6.44E-05	protein_coding
ENSG00000165389	**SPTSSA**	-0.524	6.53E-05	protein_coding
ENSG00000123080	**CDKN2C**	-0.530	6.82E-05	protein_coding
ENSG00000096150	**RPS18**	-1.221	6.93E-05	protein_coding
ENSG00000261739	**GOLGA8S**	1.220	7.67E-05	protein_coding
ENSG00000146540	**C7orf50**	-0.577	7.78E-05	protein_coding
ENSG00000204681	**GABBR1**	1.259	7.83E-05	protein_coding
ENSG00000160285	**LSS**	0.893	7.89E-05	protein_coding
ENSG00000108826	**MRPL27**	-0.735	7.95E-05	protein_coding
ENSG00000172197	**MBOAT1**	0.927	8.13E-05	protein_coding
ENSG00000132661	**NXT1**	-0.548	8.16E-05	protein_coding
ENSG00000113716	**HMGXB3**	0.596	8.22E-05	protein_coding
ENSG00000100346	**CACNA1I**	0.540	8.32E-05	protein_coding
ENSG00000162613	**FUBP1**	-1.178	8.48E-05	protein_coding
ENSG00000132694	**ARHGEF11**	0.748	8.48E-05	protein_coding
ENSG00000125459	**MSTO1**	0.560	8.61E-05	protein_coding
ENSG00000152404	**CWF19L2**	-0.512	8.61E-05	protein_coding

ENSG00000219545	**UMAD1**	-0.604	8.93E-05	protein_coding
ENSG00000269858	**EGLN2**	-0.899	9.14E-05	protein_coding
ENSG00000188342	**GTF2F2**	-0.535	9.22E-05	protein_coding
ENSG00000101464	**PIGU**	-0.525	9.45E-05	protein_coding
ENSG00000143207	**COP1**	-0.721	9.51E-05	protein_coding
ENSG00000196544	**BORCS6**	-0.631	9.81E-05	protein_coding
ENSG00000186104	**CYP2R1**	-0.739	9.85E-05	protein_coding
ENSG00000170855	**TRIAP1**	-0.500	9.93E-05	protein_coding
ENSG00000122406	**RPL5**	-1.037	1.00E-04	protein_coding
ENSG00000152348	**ATG10**	-1.039	1.00E-04	protein_coding
ENSG00000283930	**AL117339.5**	1.193	1.06E-04	protein_coding
ENSG00000262826	**INTS3**	0.583	1.08E-04	protein_coding
ENSG00000198783	**ZNF830**	-0.741	1.10E-04	protein_coding
ENSG00000188785	**ZNF548**	0.710	1.18E-04	protein_coding
ENSG00000167895	**TMC8**	0.514	1.19E-04	protein_coding
ENSG00000107404	**DVL1**	0.533	1.20E-04	protein_coding
ENSG00000181458	**TMEM45A**	0.836	1.20E-04	protein_coding
ENSG00000169442	**CD52**	-0.588	1.23E-04	protein_coding
ENSG00000213420	**GPC2**	0.973	1.38E-04	protein_coding
ENSG00000113088	**GZMK**	-0.951	1.41E-04	protein_coding
ENSG00000103249	**CLCN7**	0.655	1.41E-04	protein_coding
ENSG00000089154	**GCN1**	0.632	1.42E-04	protein_coding
ENSG00000099949	**LZTR1**	0.616	1.42E-04	protein_coding
ENSG00000138495	**COX17**	-0.647	1.42E-04	protein_coding
ENSG00000102225	**CDK16**	0.508	1.45E-04	protein_coding
ENSG00000155868	**MED7**	-0.575	1.46E-04	protein_coding
ENSG00000152061	**RABGAP1L**	-0.526	1.50E-04	protein_coding
ENSG00000198838	**RYR3**	1.465	1.52E-04	protein_coding
ENSG00000189152	**GRAPL**	0.915	1.52E-04	protein_coding
ENSG00000138439	**FAM117B**	-0.575	1.62E-04	protein_coding
ENSG00000215472	**RPL17-C18orf32**	-0.674	1.64E-04	protein_coding
ENSG00000156931	**VPS8**	0.581	1.68E-04	protein_coding
ENSG00000182979	**MTA1**	0.548	1.81E-04	protein_coding
ENSG00000173200	**PARP15**	0.704	1.84E-04	protein_coding
ENSG00000166405	**RIC3**	0.902	1.88E-04	protein_coding
ENSG00000170049	**KCNAB3**	1.071	1.88E-04	protein_coding
ENSG00000273494	**PANK4**	0.896	1.91E-04	protein_coding
ENSG00000108515	**ENO3**	0.878	1.94E-04	protein_coding
ENSG00000008324	**SS18L2**	-0.608	1.98E-04	protein_coding
ENSG00000123545	**NDUFAF4**	-0.561	2.02E-04	protein_coding
ENSG00000137656	**BUD13**	-0.605	2.03E-04	protein_coding
ENSG00000172239	**PAIP1**	-0.602	2.03E-04	protein_coding
ENSG00000135473	**PAN2**	0.854	2.09E-04	protein_coding
ENSG00000164898	**FMC1**	-0.764	2.12E-04	protein_coding
ENSG00000121060	**TRIM25**	0.559	2.23E-04	protein_coding
ENSG00000175455	**CCDC14**	0.828	2.24E-04	protein_coding
ENSG00000278788	**SBNO2**	0.746	2.24E-04	protein_coding
ENSG00000155980	**KIF5A**	0.890	2.30E-04	protein_coding
ENSG00000140379	**BCL2A1**	-0.668	2.32E-04	protein_coding
ENSG00000027001	**MIPEP**	-0.707	2.32E-04	protein_coding

ENSG00000116198	**CEP104**	0.596	2.32E-04	protein_coding
ENSG00000102858	**MGRN1**	0.631	2.38E-04	protein_coding
ENSG00000106479	**ZNF862**	0.656	2.46E-04	protein_coding
ENSG00000165572	**KBTBD6**	-0.783	2.46E-04	protein_coding
ENSG00000129235	**TXNDC17**	-0.635	2.47E-04	protein_coding
ENSG00000151779	**NBAS**	-0.713	2.47E-04	protein_coding
ENSG00000239732	**TLR9**	1.355	2.54E-04	protein_coding
ENSG00000141012	**GALNS**	0.505	2.57E-04	protein_coding
ENSG00000285077	**AC091057.6**	0.591	2.62E-04	protein_coding
ENSG00000136828	**RALGPS1**	0.839	2.64E-04	protein_coding
ENSG00000198937	**CCDC167**	-0.716	2.68E-04	protein_coding
ENSG00000221866	**PLXNA4**	1.186	2.68E-04	protein_coding
ENSG00000132530	**XAF1**	0.590	2.74E-04	protein_coding
ENSG00000141378	**PTRH2**	-0.531	2.76E-04	protein_coding
ENSG00000167565	**SERTAD3**	-1.091	2.76E-04	protein_coding
ENSG00000105679	**GAPDHS**	1.029	2.78E-04	protein_coding
ENSG00000164967	**RPP25L**	-0.636	2.82E-04	protein_coding
ENSG00000141543	**EIF4A3**	-0.672	2.85E-04	protein_coding
ENSG00000135211	**TMEM60**	-0.617	2.90E-04	protein_coding
ENSG00000283580	**AC098484.3**	-0.639	2.91E-04	protein_coding
ENSG00000224877	**NDUFAF8**	-0.821	2.92E-04	protein_coding
ENSG00000137513	**NARS2**	-0.628	2.96E-04	protein_coding
ENSG00000156603	**MED19**	-0.657	3.03E-04	protein_coding
ENSG00000275003	**DDT**	-0.523	3.03E-04	protein_coding
ENSG00000162591	**MEGF6**	0.702	3.10E-04	protein_coding
ENSG00000104907	**TRMT1**	0.566	3.14E-04	protein_coding
ENSG00000133858	**ZFC3H1**	0.611	3.14E-04	protein_coding
ENSG00000143443	**C1orf56**	-0.874	3.14E-04	protein_coding
ENSG00000172586	**CHCHD1**	-0.633	3.14E-04	protein_coding
ENSG00000214736	**TOMM6**	-0.512	3.17E-04	protein_coding
ENSG00000099381	**SETD1A**	0.501	3.34E-04	protein_coding
ENSG00000100055	**CYTH4**	0.507	3.34E-04	protein_coding
ENSG00000154803	**FLCN**	0.568	3.34E-04	protein_coding
ENSG00000105576	**TNPO2**	0.647	3.43E-04	protein_coding
ENSG00000126749	**EMG1**	-0.650	3.46E-04	protein_coding
ENSG00000124782	**RREB1**	0.768	3.52E-04	protein_coding
ENSG00000226492	**CUTA**	-1.085	3.55E-04	protein_coding
ENSG00000083093	**PALB2**	-0.779	3.64E-04	protein_coding
ENSG00000176248	**ANAPC2**	0.565	3.74E-04	protein_coding
ENSG00000237651	**C2orf74**	-0.531	3.74E-04	protein_coding
ENSG00000275740	**AC091959.3**	1.294	3.80E-04	protein_coding
ENSG00000232112	**TMA7**	-0.630	3.81E-04	protein_coding
ENSG00000161381	**PLXDC1**	0.879	3.82E-04	protein_coding
ENSG00000143156	**NME7**	-0.616	3.95E-04	protein_coding
ENSG00000124571	**XPO5**	0.596	3.95E-04	protein_coding
ENSG00000109390	**NDUFC1**	-0.573	4.08E-04	protein_coding
ENSG00000103671	**TRIP4**	-0.521	4.17E-04	protein_coding
ENSG00000235453	**SMIM27**	-0.636	4.20E-04	protein_coding
ENSG00000139990	**DCAF5**	0.512	4.26E-04	protein_coding
ENSG00000096070	**BRPF3**	0.562	4.32E-04	protein_coding

ENSG00000114812	**VIPR1**	0.551	4.32E-04	protein_coding
ENSG00000117862	**TXNDC12**	-0.563	4.32E-04	protein_coding
ENSG00000104213	**PDGFRL**	-1.290	4.36E-04	protein_coding
ENSG00000188343	**FAM92A**	-0.829	4.36E-04	protein_coding
ENSG00000120662	**MTRF1**	0.557	4.37E-04	protein_coding
ENSG00000162104	**ADCY9**	0.653	4.37E-04	protein_coding
ENSG00000168000	**BSCL2**	-1.207	4.43E-04	protein_coding
ENSG00000188211	**NCR3LG1**	1.273	4.43E-04	protein_coding
ENSG00000205238	**SPDYE2**	0.852	4.43E-04	protein_coding
ENSG00000280670	**CCDC163**	1.045	4.43E-04	protein_coding
ENSG00000203663	**OR2L2**	0.930	4.50E-04	protein_coding
ENSG00000168137	**SETD5**	0.672	4.61E-04	protein_coding
ENSG00000161664	**ASB16**	0.819	4.71E-04	protein_coding
ENSG00000011260	**UTP18**	-0.718	4.76E-04	protein_coding
ENSG00000168056	**LTBP3**	0.665	4.76E-04	protein_coding
ENSG00000172315	**TP53RK**	-0.518	4.80E-04	protein_coding
ENSG00000145623	**OSMR**	1.258	4.85E-04	protein_coding
ENSG00000132432	**SEC61G**	-0.662	4.85E-04	protein_coding
ENSG00000152147	**GEMIN6**	-0.647	4.85E-04	protein_coding
ENSG00000108797	**CNTNAP1**	0.704	4.87E-04	protein_coding
ENSG00000148343	**MIGA2**	0.544	4.91E-04	protein_coding
ENSG00000106443	**PHF14**	-0.900	5.01E-04	protein_coding
ENSG00000123415	**SMUG1**	-0.543	5.10E-04	protein_coding
ENSG00000179271	**GADD45GIP1**	-0.667	5.12E-04	protein_coding
ENSG00000197283	**SYNGAP1**	1.191	5.16E-04	protein_coding
ENSG00000198746	**GPATCH3**	-0.780	5.16E-04	protein_coding
ENSG00000204152	**TIMM23B**	0.516	5.16E-04	protein_coding
ENSG00000146021	**KLHL3**	0.689	5.21E-04	protein_coding
ENSG00000103064	**SLC7A6**	0.592	5.26E-04	protein_coding
ENSG00000009694	**TENM1**	0.887	5.34E-04	protein_coding
ENSG00000013441	**CLK1**	-1.003	5.34E-04	protein_coding
ENSG00000164306	**PRIMPOL**	-0.633	5.34E-04	protein_coding
ENSG00000196557	**CACNA1H**	0.714	5.34E-04	protein_coding
ENSG00000182700	**IGIP**	-0.635	5.36E-04	protein_coding
ENSG00000092531	**SNAP23**	0.904	5.40E-04	protein_coding
ENSG00000262919	**CCNQ**	-0.522	5.40E-04	protein_coding
ENSG00000107018	**RLN1**	-0.676	5.42E-04	protein_coding
ENSG00000053438	**NNAT**	1.268	5.45E-04	protein_coding
ENSG00000205323	**SARNP**	-0.561	5.59E-04	protein_coding
ENSG00000253304	**TMEM200B**	-1.175	5.62E-04	protein_coding
ENSG00000106588	**PSMA2**	-0.555	5.63E-04	protein_coding
ENSG00000275145	**FRG1**	-0.944	5.64E-04	protein_coding
ENSG00000113369	**ARRDC3**	0.845	5.80E-04	protein_coding
ENSG00000186395	**KRT10**	-0.623	5.83E-04	protein_coding
ENSG00000100393	**EP300**	0.726	5.90E-04	protein_coding
ENSG00000113108	**APBB3**	0.694	5.91E-04	protein_coding
ENSG00000152556	**PFKM**	0.528	5.93E-04	protein_coding
ENSG00000037757	**MRI1**	0.597	5.99E-04	protein_coding
ENSG00000116001	**TIA1**	0.562	6.01E-04	protein_coding
ENSG00000070501	**POLB**	-0.722	6.01E-04	protein_coding

ENSG00000105245	**NUMBL**	0.563	6.04E-04	protein_coding
ENSG00000141293	**SKAP1**	-0.657	6.04E-04	protein_coding
ENSG00000274081	**PUF60**	-0.672	6.08E-04	protein_coding
ENSG00000159063	**ALG8**	-0.526	6.25E-04	protein_coding
ENSG00000281950	**SPDYE16**	0.573	6.25E-04	protein_coding
ENSG00000064102	**INTS13**	-0.585	6.56E-04	protein_coding
ENSG00000154760	**SLFN13**	0.638	6.56E-04	protein_coding
ENSG00000115568	**ZNF142**	0.641	6.69E-04	protein_coding
ENSG00000127922	**SEM1**	-0.515	6.73E-04	protein_coding
ENSG00000168259	**DNAJC7**	-0.674	6.73E-04	protein_coding
ENSG00000111886	**GABRR2**	1.350	6.90E-04	protein_coding
ENSG00000142694	**EVA1B**	-0.757	6.90E-04	protein_coding
ENSG00000197879	**MYO1C**	0.540	6.90E-04	protein_coding
ENSG00000227507	**LTB**	-1.133	6.90E-04	protein_coding
ENSG00000170417	**TMEM182**	0.857	6.92E-04	protein_coding
ENSG00000221909	**FAM200A**	-0.562	7.05E-04	protein_coding
ENSG00000165501	**LRR1**	-0.532	7.29E-04	protein_coding
ENSG00000173915	**ATP5MD**	-0.603	7.37E-04	protein_coding
ENSG00000178718	**RPP25**	-0.547	7.58E-04	protein_coding
ENSG00000143442	**POGZ**	0.602	7.58E-04	protein_coding
ENSG00000187764	**SEMA4D**	0.554	7.65E-04	protein_coding
ENSG00000185621	**LMLN**	0.606	7.76E-04	protein_coding
ENSG00000185669	**SNAI3**	-0.577	7.83E-04	protein_coding
ENSG00000203778	**FAM229B**	-0.790	7.99E-04	protein_coding
ENSG00000139187	**KLRG1**	-0.626	8.06E-04	protein_coding
ENSG00000262418	**PTPRC**	1.002	8.43E-04	protein_coding
ENSG00000100258	**LMF2**	0.600	8.50E-04	protein_coding
ENSG00000269343	**ZNF587B**	0.665	8.53E-04	protein_coding
ENSG00000131495	**NDUFA2**	-0.549	8.62E-04	protein_coding
ENSG00000121716	**PILRB**	0.618	8.73E-04	protein_coding
ENSG00000184076	**UQCR10**	-0.518	8.92E-04	protein_coding
ENSG00000179094	**AC129492.1**	0.929	9.00E-04	protein_coding
ENSG00000132768	**DPH2**	0.530	9.10E-04	protein_coding
ENSG00000184281	**TSSC4**	-0.779	9.10E-04	protein_coding
ENSG00000151366	**NDUFC2**	-0.550	9.20E-04	protein_coding
ENSG00000099814	**CEP170B**	0.650	9.42E-04	protein_coding
ENSG00000183648	**NDUFB1**	-0.740	9.46E-04	protein_coding
ENSG00000145649	**GZMA**	-0.900	9.69E-04	protein_coding
ENSG00000270231	**NBPF8**	0.677	9.92E-04	protein_coding
ENSG00000068650	**ATP11A**	0.801	9.95E-04	protein_coding
ENSG00000134480	**CCNH**	-0.629	9.95E-04	protein_coding
ENSG00000115484	**CCT4**	-0.663	9.98E-04	protein_coding
ENSG00000124217	**MOCS3**	-0.513	1.00E-03	protein_coding
ENSG00000127774	**EMC6**	-0.521	1.01E-03	protein_coding
ENSG00000163806	**SPDYA**	-1.283	1.01E-03	protein_coding
ENSG00000167766	**ZNF83**	0.643	1.02E-03	protein_coding
ENSG00000107736	**CDH23**	0.578	1.04E-03	protein_coding
ENSG00000154723	**ATP5PF**	-0.567	1.04E-03	protein_coding
ENSG00000115904	**SOS1**	0.713	1.05E-03	protein_coding
ENSG00000129473	**BCL2L2**	0.832	1.05E-03	protein_coding

ENSG00000112667	**DNPH1**	-0.518	1.07E-03	protein_coding
ENSG00000095319	**NUP188**	0.501	1.08E-03	protein_coding
ENSG00000100902	**PSMA6**	-0.545	1.08E-03	protein_coding
ENSG00000156030	**ELMSAN1**	0.540	1.09E-03	protein_coding
ENSG00000167702	**KIFC2**	0.782	1.09E-03	protein_coding
ENSG00000145217	**SLC26A1**	1.092	1.09E-03	protein_coding
ENSG00000090238	**YPEL3**	-0.613	1.09E-03	protein_coding
ENSG00000056050	**HPF1**	-0.582	1.09E-03	protein_coding
ENSG00000255508	**AP002990.1**	-1.122	1.09E-03	protein_coding
ENSG00000164919	**COX6C**	-0.528	1.09E-03	protein_coding
ENSG00000135045	**C9orf40**	-0.575	1.10E-03	protein_coding
ENSG00000092108	**SCFD1**	-0.563	1.10E-03	protein_coding
ENSG00000197019	**SERTAD1**	-0.820	1.10E-03	protein_coding
ENSG00000100387	**RBX1**	-0.509	1.10E-03	protein_coding
ENSG00000173621	**LRFN4**	0.829	1.11E-03	protein_coding
ENSG00000168255	**POLR2J3**	0.712	1.11E-03	protein_coding
ENSG00000187608	**ISG15**	-0.575	1.11E-03	protein_coding
ENSG00000243927	**MRPS6**	-0.524	1.11E-03	protein_coding
ENSG00000211450	**SELENOH**	-0.654	1.12E-03	protein_coding
ENSG00000166783	**MARF1**	-0.590	1.12E-03	protein_coding
ENSG00000179588	**ZFPM1**	0.701	1.12E-03	protein_coding
ENSG00000166046	**TCP11L2**	1.023	1.13E-03	protein_coding
ENSG00000072274	**TFRC**	0.567	1.13E-03	protein_coding
ENSG00000168701	**TMEM208**	-0.678	1.14E-03	protein_coding
ENSG00000250151	**ARPC4-TTLL3**	0.963	1.14E-03	protein_coding
ENSG00000285269	**AL160269.1**	1.007	1.16E-03	protein_coding
ENSG00000163154	**TNFAIP8L2**	-0.881	1.17E-03	protein_coding
ENSG00000156050	**FAM161B**	-1.243	1.19E-03	protein_coding
ENSG00000004779	**NDUFAB1**	-0.535	1.21E-03	protein_coding
ENSG00000134248	**LAMTOR5**	-0.501	1.21E-03	protein_coding
ENSG00000176658	**MYO1D**	0.768	1.21E-03	protein_coding
ENSG00000164142	**FAM160A1**	-1.216	1.22E-03	protein_coding
ENSG00000023572	**GLRX2**	-0.571	1.22E-03	protein_coding
ENSG00000150779	**TIMM8B**	-0.673	1.22E-03	protein_coding
ENSG00000227801	**COL11A2**	0.799	1.23E-03	protein_coding
ENSG00000095587	**TLL2**	0.894	1.23E-03	protein_coding
ENSG00000160973	**FOXH1**	-0.602	1.26E-03	protein_coding
ENSG00000198258	**UBL5**	-0.552	1.29E-03	protein_coding
ENSG00000096433	**ITPR3**	0.720	1.30E-03	protein_coding
ENSG00000148834	**GSTO1**	-0.567	1.30E-03	protein_coding
ENSG00000092330	**AL096870.1**	-0.570	1.30E-03	protein_coding
ENSG00000171763	**SPATA5L1**	-0.608	1.31E-03	protein_coding
ENSG00000108556	**CHRNE**	-1.173	1.31E-03	protein_coding
ENSG00000132963	**POMP**	-0.508	1.31E-03	protein_coding
ENSG00000105617	**LENG1**	-0.885	1.33E-03	protein_coding
ENSG00000155561	**NUP205**	-0.657	1.37E-03	protein_coding
ENSG00000118260	**CREB1**	0.671	1.39E-03	protein_coding
ENSG00000181029	**TRAPPC5**	-0.702	1.39E-03	protein_coding
ENSG00000143797	**MBOAT2**	0.829	1.40E-03	protein_coding
ENSG00000140471	**LINS1**	0.615	1.43E-03	protein_coding

ENSG00000204176	**SYT15**	-0.808	1.43E-03	protein_coding
ENSG00000137504	**CREBZF**	0.567	1.44E-03	protein_coding
ENSG00000146232	**NFKBIE**	-0.771	1.44E-03	protein_coding
ENSG00000175198	**PCCA**	-0.716	1.45E-03	protein_coding
ENSG00000155506	**LARP1**	0.516	1.46E-03	protein_coding
ENSG00000004777	**ARHGAP33**	1.025	1.47E-03	protein_coding
ENSG00000169020	**ATP5ME**	-0.731	1.47E-03	protein_coding
ENSG00000181191	**PJA1**	-0.642	1.47E-03	protein_coding
ENSG00000267680	**ZNF224**	0.546	1.48E-03	protein_coding
ENSG00000110944	**IL23A**	-0.522	1.49E-03	protein_coding
ENSG00000151694	**ADAM17**	0.570	1.52E-03	protein_coding
ENSG00000106245	**BUD31**	-0.556	1.52E-03	protein_coding
ENSG00000172172	**MRPL13**	-0.754	1.52E-03	protein_coding
ENSG00000103507	**BCKDK**	-0.638	1.52E-03	protein_coding
ENSG00000196967	**ZNF585A**	-0.547	1.52E-03	protein_coding
ENSG00000169246	**NPIPB3**	0.900	1.56E-03	protein_coding
ENSG00000119705	**SLIRP**	-0.581	1.57E-03	protein_coding
ENSG00000049449	**RCN1**	-0.784	1.59E-03	protein_coding
ENSG00000109685	**NSD2**	0.546	1.59E-03	protein_coding
ENSG00000089041	**P2RX7**	0.619	1.59E-03	protein_coding
ENSG00000156411	**ATP5MPL**	-0.634	1.59E-03	protein_coding
ENSG00000053702	**NRIP2**	0.625	1.61E-03	protein_coding
ENSG00000205649	**HTN3**	1.008	1.62E-03	protein_coding
ENSG00000228486	**C2orf92**	0.758	1.64E-03	protein_coding
ENSG00000122705	**CLTA**	-0.558	1.64E-03	protein_coding
ENSG00000168275	**COA6**	-0.505	1.65E-03	protein_coding
ENSG00000183978	**COA3**	-0.628	1.65E-03	protein_coding
ENSG00000082258	**CCNT2**	0.569	1.68E-03	protein_coding
ENSG00000108651	**UTP6**	-0.501	1.69E-03	protein_coding
ENSG00000148832	**PAOX**	-0.827	1.72E-03	protein_coding
ENSG00000130755	**GMFG**	-0.524	1.73E-03	protein_coding
ENSG00000131966	**ACTR10**	-0.519	1.76E-03	protein_coding
ENSG00000162227	**TAF6L**	0.542	1.82E-03	protein_coding
ENSG00000167861	**HID1**	0.610	1.87E-03	protein_coding
ENSG00000168229	**PTGDR**	-0.819	1.90E-03	protein_coding
ENSG00000126264	**HCST**	-0.559	1.91E-03	protein_coding
ENSG00000206560	**ANKRD28**	0.583	1.91E-03	protein_coding
ENSG00000259112	**NDUFC2-KCTD14**	-0.683	1.91E-03	protein_coding
ENSG00000171806	**METTL18**	-0.585	1.91E-03	protein_coding
ENSG00000175193	**PARL**	-0.775	1.92E-03	protein_coding
ENSG00000242485	**MRPL20**	-0.503	1.92E-03	protein_coding
ENSG00000118369	**USP35**	0.574	1.92E-03	protein_coding
ENSG00000120451	**SNX19**	0.538	1.92E-03	protein_coding
ENSG00000175066	**GK5**	0.766	1.92E-03	protein_coding
ENSG00000281991	**TMEM265**	-1.047	1.93E-03	protein_coding
ENSG00000007923	**DNAJC11**	0.540	1.95E-03	protein_coding
ENSG00000243449	**C4orf48**	-0.818	1.96E-03	protein_coding
ENSG00000225697	**SLC26A6**	0.630	2.01E-03	protein_coding
ENSG00000163584	**RPL22L1**	-0.627	2.05E-03	protein_coding
ENSG00000130803	**ZNF317**	-0.759	2.09E-03	protein_coding

ENSG00000172428	**COPS9**	-0.678	2.09E-03	protein_coding
ENSG00000153395	**LPCAT1**	-0.657	2.12E-03	protein_coding
ENSG00000281406	**BLACAT1**	-1.230	2.12E-03	protein_coding
ENSG00000205544	**TMEM256**	-0.650	2.13E-03	protein_coding
ENSG00000095713	**CRTAC1**	1.183	2.14E-03	protein_coding
ENSG00000130758	**MAP3K10**	0.560	2.15E-03	protein_coding
ENSG00000137133	**HINT2**	-0.595	2.20E-03	protein_coding
ENSG00000107371	**EXOSC3**	-0.672	2.20E-03	protein_coding
ENSG00000213918	**DNASE1**	0.503	2.20E-03	protein_coding
ENSG00000181982	**CCDC149**	1.145	2.22E-03	protein_coding
ENSG00000232388	**SMIM26**	-0.514	2.29E-03	protein_coding
ENSG00000130748	**TMEM160**	-0.717	2.31E-03	protein_coding
ENSG00000137038	**DMAC1**	-0.655	2.32E-03	protein_coding
ENSG00000166924	**NYAP1**	0.637	2.32E-03	protein_coding
ENSG00000179912	**R3HDM2**	0.599	2.32E-03	protein_coding
ENSG00000164610	**RP9**	-0.532	2.34E-03	protein_coding
ENSG00000133321	**PLAAT4**	-0.523	2.36E-03	protein_coding
ENSG00000224470	**ATXN1L**	0.527	2.37E-03	protein_coding
ENSG00000138399	**FASTKD1**	-0.668	2.41E-03	protein_coding
ENSG00000075239	**ACAT1**	-0.628	2.43E-03	protein_coding
ENSG00000129083	**COPB1**	-0.546	2.45E-03	protein_coding
ENSG00000066294	**CD84**	0.744	2.45E-03	protein_coding
ENSG00000240303	**ACAD11**	0.529	2.45E-03	protein_coding
ENSG00000119737	**GPR75**	0.959	2.46E-03	protein_coding
ENSG00000069869	**NEDD4**	0.754	2.47E-03	protein_coding
ENSG00000116754	**SRSF11**	0.508	2.50E-03	protein_coding
ENSG00000118503	**TNFAIP3**	0.818	2.50E-03	protein_coding
ENSG00000148803	**FUOM**	-0.640	2.50E-03	protein_coding
ENSG00000165995	**CACNB2**	0.750	2.50E-03	protein_coding
ENSG00000146094	**DOK3**	0.818	2.50E-03	protein_coding
ENSG00000156194	**PPEF2**	1.119	2.51E-03	protein_coding
ENSG00000258461	**AC012651.1**	0.677	2.53E-03	protein_coding
ENSG00000139344	**AMDHD1**	0.736	2.56E-03	protein_coding
ENSG00000114857	**NKTR**	0.694	2.57E-03	protein_coding
ENSG00000139163	**ETNK1**	-0.503	2.58E-03	protein_coding
ENSG00000170734	**POLH**	0.549	2.60E-03	protein_coding
ENSG00000160050	**CCDC28B**	-0.644	2.62E-03	protein_coding
ENSG00000111639	**MRPL51**	-0.591	2.62E-03	protein_coding
ENSG00000154059	**IMPACT**	-0.535	2.62E-03	protein_coding
ENSG00000072840	**EVC**	0.738	2.63E-03	protein_coding
ENSG00000089692	**LAG3**	-0.775	2.63E-03	protein_coding
ENSG00000121940	**CLCC1**	-0.900	2.64E-03	protein_coding
ENSG00000176731	**RBIS**	-0.566	2.64E-03	protein_coding
ENSG00000007545	**CRAMP1**	0.555	2.67E-03	protein_coding
ENSG00000143977	**SNRPG**	-0.504	2.69E-03	protein_coding
ENSG00000168350	**DEGS2**	-1.213	2.69E-03	protein_coding
ENSG00000178567	**EPM2AIP1**	0.615	2.72E-03	protein_coding
ENSG00000067798	**NAV3**	0.839	2.73E-03	protein_coding
ENSG00000100129	**EIF3L**	-0.603	2.76E-03	protein_coding
ENSG00000125995	**ROMO1**	-0.620	2.77E-03	protein_coding

ENSG00000164615	**CAMLG**	-0.513	2.78E-03	protein_coding
ENSG00000175265	**GOLGA8A**	0.820	2.78E-03	protein_coding
ENSG00000163864	**NMNAT3**	0.707	2.81E-03	protein_coding
ENSG00000102900	**NUP93**	-0.613	2.83E-03	protein_coding
ENSG00000138095	**LRPPRC**	-0.567	2.83E-03	protein_coding
ENSG00000090615	**GOLGA3**	0.517	2.85E-03	protein_coding
ENSG00000179085	**DPM3**	-0.638	2.85E-03	protein_coding
ENSG00000139218	**SCAF11**	-0.620	2.86E-03	protein_coding
ENSG00000113448	**PDE4D**	0.748	2.88E-03	protein_coding
ENSG00000131174	**COX7B**	-0.550	2.89E-03	protein_coding
ENSG00000231377	**DHX16**	1.075	2.89E-03	protein_coding
ENSG00000171103	**TRMT61B**	-0.543	2.94E-03	protein_coding
ENSG00000137288	**UQCC2**	-0.549	2.95E-03	protein_coding
ENSG00000046653	**GPM6B**	1.006	2.97E-03	protein_coding
ENSG00000073578	**SDHA**	-0.612	2.99E-03	protein_coding
ENSG00000076351	**SLC46A1**	-0.644	2.99E-03	protein_coding
ENSG00000113621	**TXNDC15**	-0.623	3.02E-03	protein_coding
ENSG00000188878	**FBF1**	0.551	3.02E-03	protein_coding
ENSG00000164620	**RELL2**	0.500	3.05E-03	protein_coding
ENSG00000177191	**B3GNT8**	-1.055	3.06E-03	protein_coding
ENSG00000198740	**ZNF652**	-0.659	3.07E-03	protein_coding
ENSG00000067191	**CACNB1**	0.605	3.12E-03	protein_coding
ENSG00000130699	**TAF4**	0.981	3.13E-03	protein_coding
ENSG00000213171	**LINGO4**	1.078	3.13E-03	protein_coding
ENSG00000179144	**GIMAP7**	-0.664	3.13E-03	protein_coding
ENSG00000182087	**TMEM259**	0.564	3.18E-03	protein_coding
ENSG00000185670	**ZBTB3**	-0.766	3.18E-03	protein_coding
ENSG00000198720	**ANKRD13B**	0.750	3.19E-03	protein_coding
ENSG00000204922	**UQCC3**	-0.577	3.21E-03	protein_coding
ENSG00000178449	**COX14**	-0.593	3.22E-03	protein_coding
ENSG00000104450	**SPAG1**	0.826	3.23E-03	protein_coding
ENSG00000177000	**MTHFR**	0.550	3.27E-03	protein_coding
ENSG00000147324	**MFHAS1**	0.540	3.28E-03	protein_coding
ENSG00000183336	**BOLA2**	-0.518	3.32E-03	protein_coding
ENSG00000005075	**POLR2J**	-0.576	3.33E-03	protein_coding
ENSG00000185088	**RPS27L**	-0.593	3.34E-03	protein_coding
ENSG00000088970	**KIZ**	-1.006	3.35E-03	protein_coding
ENSG00000060688	**SNRNP40**	-0.505	3.39E-03	protein_coding
ENSG00000099795	**NDUFB7**	-0.527	3.41E-03	protein_coding
ENSG00000135441	**BLOC1S1**	-0.506	3.41E-03	protein_coding
ENSG00000173480	**ZNF417**	-0.659	3.41E-03	protein_coding
ENSG00000092020	**PPP2R3C**	-0.586	3.45E-03	protein_coding
ENSG00000169508	**GPR183**	-0.527	3.45E-03	protein_coding
ENSG00000243156	**MICAL3**	0.579	3.48E-03	protein_coding
ENSG00000100815	**TRIP11**	0.572	3.48E-03	protein_coding
ENSG00000132300	**PTCD3**	0.905	3.49E-03	protein_coding
ENSG00000075624	**ACTB**	-0.592	3.60E-03	protein_coding
ENSG00000162576	**MXRA8**	0.995	3.61E-03	protein_coding
ENSG00000278615	**C11orf98**	-0.592	3.62E-03	protein_coding
ENSG00000263290	**SCAMP3**	-0.807	3.63E-03	protein_coding

ENSG00000127418	**FGFRL1**	0.730	3.66E-03	protein_coding
ENSG00000177600	**RPLP2**	-0.674	3.67E-03	protein_coding
ENSG00000067334	**DNTTIP2**	-0.528	3.68E-03	protein_coding
ENSG00000129219	**PLD2**	0.655	3.68E-03	protein_coding
ENSG00000085998	**POMGNT1**	-0.828	3.73E-03	protein_coding
ENSG00000117691	**NENF**	-0.636	3.73E-03	protein_coding
ENSG00000185324	**CDK10**	0.760	3.73E-03	protein_coding
ENSG00000274233	**CCL5**	-0.639	3.73E-03	protein_coding
ENSG00000148053	**NTRK2**	0.934	3.73E-03	protein_coding
ENSG00000169413	**RNASE6**	-0.632	3.78E-03	protein_coding
ENSG00000101654	**RNMT**	-0.560	3.82E-03	protein_coding
ENSG00000167080	**B4GALNT2**	0.768	3.83E-03	protein_coding
ENSG00000213020	**ZNF611**	0.535	3.83E-03	protein_coding
ENSG00000183878	**UTY**	0.680	3.84E-03	protein_coding
ENSG00000188958	**UTS2B**	0.863	3.84E-03	protein_coding
ENSG00000205220	**PSMB10**	-0.592	3.84E-03	protein_coding
ENSG00000143353	**LYPLAL1**	-0.555	3.86E-03	protein_coding
ENSG00000198918	**RPL39**	-0.703	3.89E-03	protein_coding
ENSG00000280663	**PCMTD2**	0.981	3.93E-03	protein_coding
ENSG00000168061	**SAC3D1**	-0.573	3.93E-03	protein_coding
ENSG00000142303	**ADAMTS10**	0.745	3.94E-03	protein_coding
ENSG00000175602	**CCDC85B**	-0.570	3.94E-03	protein_coding
ENSG00000258315	**C17orf49**	-0.561	3.94E-03	protein_coding
ENSG00000112715	**VEGFA**	1.127	3.96E-03	protein_coding
ENSG00000284454	**AC256236.3**	0.726	3.96E-03	protein_coding
ENSG00000125877	**ITPA**	-0.595	3.99E-03	protein_coding
ENSG00000134444	**RELCH**	0.596	4.01E-03	protein_coding
ENSG00000108187	**PBLD**	0.555	4.02E-03	protein_coding
ENSG00000173272	**MZT2A**	-0.540	4.05E-03	protein_coding
ENSG00000176695	**AC008977.1**	0.806	4.10E-03	protein_coding
ENSG00000166398	**KIAA0355**	0.890	4.13E-03	protein_coding
ENSG00000125743	**SNRPD2**	-0.654	4.13E-03	protein_coding
ENSG00000012817	**KDM5D**	1.011	4.16E-03	protein_coding
ENSG00000182326	**C1S**	0.946	4.18E-03	protein_coding
ENSG00000158863	**FAM160B2**	0.554	4.21E-03	protein_coding
ENSG00000147883	**CDKN2B**	0.982	4.21E-03	protein_coding
ENSG00000169288	**MRPL1**	-0.563	4.27E-03	protein_coding
ENSG00000117560	**FASLG**	-0.813	4.32E-03	protein_coding
ENSG00000046604	**DSG2**	0.759	4.33E-03	protein_coding
ENSG00000127540	**UQCR11**	-0.525	4.33E-03	protein_coding
ENSG00000147162	**OGT**	0.642	4.33E-03	protein_coding
ENSG00000169252	**ADRB2**	-0.848	4.35E-03	protein_coding
ENSG00000145220	**LYAR**	-0.573	4.35E-03	protein_coding
ENSG00000197774	**EME2**	0.612	4.35E-03	protein_coding
ENSG00000188243	**COMMD6**	-0.599	4.37E-03	protein_coding
ENSG00000063438	**AHRR**	0.801	4.38E-03	protein_coding
ENSG00000141522	**ARHGDIA**	-0.676	4.38E-03	protein_coding
ENSG00000172716	**SLFN11**	0.546	4.38E-03	protein_coding
ENSG00000128536	**CDHR3**	0.506	4.42E-03	protein_coding
ENSG00000196388	**INCA1**	0.753	4.43E-03	protein_coding

ENSG00000204936	**CD177**	1.114	4.43E-03	protein_coding
ENSG00000225590	**VPS52**	0.943	4.45E-03	protein_coding
ENSG00000006015	**REX1BD**	-0.651	4.45E-03	protein_coding
ENSG00000108559	**NUP88**	-0.502	4.46E-03	protein_coding
ENSG00000125652	**ALKBH7**	-0.520	4.49E-03	protein_coding
ENSG00000103254	**FAM173A**	-0.628	4.53E-03	protein_coding
ENSG00000196411	**EPHB4**	0.709	4.58E-03	protein_coding
ENSG00000135679	**MDM2**	-0.792	4.66E-03	protein_coding
ENSG00000145945	**FAM50B**	-0.525	4.76E-03	protein_coding
ENSG00000188010	**MORN2**	-0.661	4.76E-03	protein_coding
ENSG00000130772	**MED18**	-0.629	4.77E-03	protein_coding
ENSG00000182557	**SPNS3**	-0.636	4.77E-03	protein_coding
ENSG00000167851	**CD300A**	-0.705	4.80E-03	protein_coding
ENSG00000124172	**ATP5F1E**	-0.635	4.84E-03	protein_coding
ENSG00000233493	**TMEM238**	-0.683	4.88E-03	protein_coding
ENSG00000103479	**RBL2**	0.622	4.94E-03	protein_coding
ENSG00000157168	**NRG1**	0.879	4.94E-03	protein_coding
ENSG00000185875	**THNSL1**	-0.541	4.97E-03	protein_coding
ENSG00000064489	**BORCS8-MEF2B**	0.806	5.02E-03	protein_coding
ENSG00000183011	**NAA38**	-0.671	5.03E-03	protein_coding
ENSG00000196458	**ZNF605**	0.505	5.03E-03	protein_coding
ENSG00000259030	**FPGT-TNNI3K**	1.105	5.07E-03	protein_coding
ENSG00000125898	**FAM110A**	-0.562	5.14E-03	protein_coding
ENSG00000116539	**ASH1L**	0.554	5.15E-03	protein_coding
ENSG00000178809	**TRIM73**	0.663	5.15E-03	protein_coding
ENSG00000139574	**NPFF**	-0.917	5.17E-03	protein_coding
ENSG00000146830	**GIGYF1**	0.551	5.17E-03	protein_coding
ENSG00000243056	**EIF4EBP3**	-0.693	5.17E-03	protein_coding
ENSG00000255339	**AL133352.1**	0.872	5.20E-03	protein_coding
ENSG00000102057	**KCND1**	0.746	5.22E-03	protein_coding
ENSG00000180739	**S1PR5**	-1.074	5.24E-03	protein_coding
ENSG00000154920	**EME1**	-0.838	5.26E-03	protein_coding
ENSG00000118804	**STBD1**	-0.682	5.37E-03	protein_coding
ENSG00000233927	**RPS28**	-0.663	5.38E-03	protein_coding
ENSG00000084112	**SSH1**	0.563	5.44E-03	protein_coding
ENSG00000134247	**PTGFRN**	-1.076	5.44E-03	protein_coding
ENSG00000187790	**FANCM**	-0.729	5.56E-03	protein_coding
ENSG00000178460	**MCMDC2**	-0.691	5.57E-03	protein_coding
ENSG00000077458	**FAM76B**	0.622	5.57E-03	protein_coding
ENSG00000165105	**RASEF**	0.816	5.60E-03	protein_coding
ENSG00000213906	**LTB4R2**	0.707	5.66E-03	protein_coding
ENSG00000171863	**RPS7**	-0.552	5.72E-03	protein_coding
ENSG00000170248	**PDCD6IP**	0.639	5.76E-03	protein_coding
ENSG00000284491	**THSD8**	-0.682	5.86E-03	protein_coding
ENSG00000283189	**AC104452.1**	0.788	5.87E-03	protein_coding
ENSG00000184515	**BEX5**	-0.538	5.88E-03	protein_coding
ENSG00000168394	**TAP1**	-0.550	5.91E-03	protein_coding
ENSG00000169964	**TMEM42**	-0.513	5.92E-03	protein_coding
ENSG00000258555	**PECC1L-ADORA2**	1.091	5.92E-03	protein_coding
ENSG00000270800	**RPS10-NUDT3**	-0.902	5.92E-03	protein_coding

ENSG00000188596	**CFAP54**	0.631	5.95E-03	protein_coding
ENSG00000185379	**RAD51D**	0.861	5.97E-03	protein_coding
ENSG00000008382	**MPND**	-0.706	6.04E-03	protein_coding
ENSG00000164283	**ESM1**	-0.827	6.08E-03	protein_coding
ENSG00000157110	**RBPMS**	0.738	6.20E-03	protein_coding
ENSG00000274808	**TBC1D3B**	1.029	6.21E-03	protein_coding
ENSG00000111816	**FRK**	0.670	6.22E-03	protein_coding
ENSG00000232119	**MCTS1**	-0.574	6.22E-03	protein_coding
ENSG00000120696	**KBTBD7**	-0.637	6.24E-03	protein_coding
ENSG00000129158	**SERGEF**	-0.562	6.30E-03	protein_coding
ENSG00000250479	**CHCHD10**	-0.651	6.32E-03	protein_coding
ENSG00000104361	**NIPAL2**	0.772	6.33E-03	protein_coding
ENSG00000185231	**MC2R**	1.106	6.34E-03	protein_coding
ENSG00000286237	**RMCX5-GPRASP**	0.867	6.38E-03	protein_coding
ENSG00000156218	**ADAMTSL3**	0.884	6.39E-03	protein_coding
ENSG00000015532	**XYLT2**	-0.761	6.40E-03	protein_coding
ENSG00000267179	**AC008770.2**	-1.111	6.45E-03	protein_coding
ENSG00000080493	**SLC4A4**	-0.803	6.49E-03	protein_coding
ENSG00000065833	**ME1**	-1.064	6.52E-03	protein_coding
ENSG00000213145	**CRIP1**	-0.532	6.53E-03	protein_coding
ENSG00000189129	**PLAC9**	0.956	6.61E-03	protein_coding
ENSG00000134809	**TIMM10**	-0.560	6.65E-03	protein_coding
ENSG00000033122	**LRRC7**	1.008	6.67E-03	protein_coding
ENSG00000177156	**TALDO1**	-0.649	6.67E-03	protein_coding
ENSG00000168502	**MTCL1**	1.051	6.68E-03	protein_coding
ENSG00000270617	**URGCP-MRPS24**	-0.748	6.70E-03	protein_coding
ENSG00000164405	**UQCRQ**	-0.550	6.73E-03	protein_coding
ENSG00000196776	**CD47**	-0.567	6.73E-03	protein_coding
ENSG00000173517	**PEAK1**	0.750	6.75E-03	protein_coding
ENSG00000095539	**SEMA4G**	0.891	6.75E-03	protein_coding
ENSG00000164338	**UTP15**	0.531	6.75E-03	protein_coding
ENSG00000134909	**ARHGAP32**	0.687	6.78E-03	protein_coding
ENSG00000141527	**CARD14**	0.597	6.82E-03	protein_coding
ENSG00000176014	**TUBB6**	-1.102	6.82E-03	protein_coding
ENSG00000121578	**B4GALT4**	-0.589	6.84E-03	protein_coding
ENSG00000123144	**TRIR**	-0.502	6.87E-03	protein_coding
ENSG00000074071	**MRPS34**	-0.512	6.90E-03	protein_coding
ENSG00000100033	**PRODH**	0.681	7.00E-03	protein_coding
ENSG00000215252	**GOLGA8B**	0.809	7.02E-03	protein_coding
ENSG00000005302	**MSL3**	-0.572	7.09E-03	protein_coding
ENSG00000174099	**MSRB3**	0.681	7.14E-03	protein_coding
ENSG00000133019	**CHRM3**	-0.976	7.20E-03	protein_coding
ENSG00000188186	**LAMTOR4**	-0.582	7.23E-03	protein_coding
ENSG00000123179	**EBPL**	-0.514	7.25E-03	protein_coding
ENSG00000119203	**CPSF3**	-0.513	7.32E-03	protein_coding
ENSG00000152049	**KCNE4**	0.779	7.32E-03	protein_coding
ENSG00000214226	**C17orf67**	-0.694	7.32E-03	protein_coding
ENSG00000187961	**KLHL17**	0.604	7.34E-03	protein_coding
ENSG00000049860	**HEXB**	-0.534	7.41E-03	protein_coding
ENSG00000114841	**DNAH1**	0.581	7.47E-03	protein_coding

ENSG00000138326	**RPS24**	-0.552	7.49E-03	protein_coding
ENSG00000121989	**ACVR2A**	0.532	7.59E-03	protein_coding
ENSG00000104689	**TNFRSF10A**	0.532	7.63E-03	protein_coding
ENSG00000178896	**EXOSC4**	-0.509	7.65E-03	protein_coding
ENSG00000231861	**OR5K2**	1.009	7.68E-03	protein_coding
ENSG00000136514	**RTP4**	-0.595	7.73E-03	protein_coding
ENSG00000175595	**ERCC4**	0.535	7.82E-03	protein_coding
ENSG00000117318	**ID3**	-0.794	7.83E-03	protein_coding
ENSG00000136542	**GALNT5**	0.951	7.83E-03	protein_coding
ENSG00000169071	**ROR2**	-0.827	7.83E-03	protein_coding
ENSG00000175701	**MTLN**	-0.531	7.90E-03	protein_coding
ENSG00000152082	**MZT2B**	-0.540	8.04E-03	protein_coding
ENSG00000138080	**EMILIN1**	-0.589	8.04E-03	protein_coding
ENSG00000118640	**VAMP8**	-0.534	8.07E-03	protein_coding
ENSG00000182472	**CAPN12**	-0.894	8.07E-03	protein_coding
ENSG00000275565	**ALOX5**	0.904	8.09E-03	protein_coding
ENSG00000262446	**GBA**	-1.057	8.10E-03	protein_coding
ENSG00000166164	**BRD7**	-0.521	8.17E-03	protein_coding
ENSG00000125910	**S1PR4**	-0.503	8.33E-03	protein_coding
ENSG00000277140	**MARF1**	0.750	8.41E-03	protein_coding
ENSG00000002016	**RAD52**	0.567	8.44E-03	protein_coding
ENSG00000034677	**RNF19A**	0.523	8.44E-03	protein_coding
ENSG00000176853	**FAM91A1**	0.541	8.51E-03	protein_coding
ENSG00000241685	**ARPC1A**	-0.580	8.54E-03	protein_coding
ENSG00000265681	**RPL17**	-0.583	8.58E-03	protein_coding
ENSG00000174255	**ZNF80**	0.596	8.58E-03	protein_coding
ENSG00000151338	**MIPOL1**	0.716	8.62E-03	protein_coding
ENSG00000179454	**KLHL28**	0.586	8.70E-03	protein_coding
ENSG00000258311	**AC009779.3**	0.645	8.72E-03	protein_coding
ENSG00000082438	**COBLL1**	1.013	8.73E-03	protein_coding
ENSG00000166823	**MESP1**	-1.028	8.73E-03	protein_coding
ENSG00000106355	**LSM5**	-0.532	8.74E-03	protein_coding
ENSG00000083807	**SLC27A5**	-0.532	8.77E-03	protein_coding
ENSG00000187808	**SOWAHD**	-0.760	8.77E-03	protein_coding
ENSG00000283984	**AL593848.2**	-1.069	8.81E-03	protein_coding
ENSG00000146250	**PRSS35**	-1.068	8.95E-03	protein_coding
ENSG00000109475	**RPL34**	-0.570	9.01E-03	protein_coding
ENSG00000154099	**DNAAF1**	-0.966	9.01E-03	protein_coding
ENSG00000262209	**PCDHGB3**	1.039	9.04E-03	protein_coding
ENSG00000198870	**STKLD1**	-1.028	9.07E-03	protein_coding
ENSG00000102886	**GDPD3**	-0.962	9.14E-03	protein_coding
ENSG00000133863	**TEX15**	0.784	9.15E-03	protein_coding
ENSG00000174527	**MYO1H**	0.939	9.24E-03	protein_coding
ENSG00000176563	**CNTD1**	-1.009	9.26E-03	protein_coding
ENSG00000123975	**CKS2**	-0.604	9.35E-03	protein_coding
ENSG00000118263	**KLF7**	0.635	9.41E-03	protein_coding
ENSG00000135740	**SLC9A5**	0.844	9.42E-03	protein_coding
ENSG00000174842	**GLMN**	-0.579	9.43E-03	protein_coding
ENSG00000150477	**KIAA1328**	0.550	9.44E-03	protein_coding
ENSG00000079999	**KEAP1**	-0.575	9.44E-03	protein_coding

ENSG00000133138	**TBC1D8B**	0.768	9.45E-03	protein_coding
ENSG00000126883	**NUP214**	-0.669	9.47E-03	protein_coding
ENSG00000169738	**DCXR**	-0.624	9.50E-03	protein_coding
ENSG00000198752	**CDC42BPB**	0.598	9.58E-03	protein_coding
ENSG00000132792	**CTNNBL1**	-0.632	9.59E-03	protein_coding
ENSG00000116032	**GRIN3B**	0.966	9.65E-03	protein_coding
ENSG00000154025	**SLC5A10**	-0.858	9.66E-03	protein_coding
ENSG00000123349	**PFDN5**	-0.620	9.66E-03	protein_coding
ENSG00000106211	**HSPB1**	-0.595	9.73E-03	protein_coding
ENSG00000133962	**CATSPERB**	-0.937	9.75E-03	protein_coding
ENSG00000156467	**UQCRB**	-0.508	9.75E-03	protein_coding
ENSG00000198848	**CES1**	-0.872	9.77E-03	protein_coding
ENSG00000186329	**TMEM212**	0.654	9.78E-03	protein_coding
ENSG00000090266	**NDUFB2**	-0.510	9.79E-03	protein_coding
ENSG00000185420	**SMYD3**	-0.579	9.79E-03	protein_coding
ENSG00000145425	**RPS3A**	-0.522	9.80E-03	protein_coding
ENSG00000158716	**DUSP23**	-0.515	9.81E-03	protein_coding
ENSG00000159231	**CBR3**	-0.612	9.83E-03	protein_coding
ENSG00000062524	**LTK**	0.715	9.93E-03	protein_coding
ENSG00000141194	**OR4D1**	-0.517	9.93E-03	protein_coding
ENSG00000263001	**GTF2I**	0.582	9.96E-03	protein_coding
ENSG00000063180	**CA11**	-0.564	9.97E-03	protein_coding
ENSG00000132589	**FLOT2**	-0.776	9.97E-03	protein_coding

Table S2. In HS skin lesions, the transcripts corresponding to up- and down-regulated DETs of blood HS Th_{mem} cells are not more frequently up- and down-regulated than the totality of protein-coding transcripts. DETs showing no basic expression in HS skin were not included in this analysis.

	Transcriptional regulation in HS skin						
	up-regulation		no regulation		down-regulation		***P*-value**
	number	proportion	number	proportion	number	proportion	X^2 test, compared to all protein-coding transcripts
**all protein-coding transcripts (HS skin) (n = 16,351)**	2536	15.5 %	11,748	71.8 %	2031	12.4 %	-
**DETs up-regulated in HS blood Th_{mem} cells (n = 362)**	53	14,6 %	271	74.9 %	38	10.5 %	**n.s.**
**DETs down-regulated in HS blood Th_{mem} cells (n = 358)**	47	13.1 %	298	83.2 %	13	3.6 %	**< 0.001**

Table S3. The proportions of Th_{mem} cells before and after MACS^{TM}-based isolation are given as mean ± SEM (range). HS, hidradenitis suppurativa

	**proportion of Th_{mem} cells**	
	before isolation	after isolation
**Healthy**	10.40 ± 1.31<br>(6.11 – 19.49)	97.63 ± 0.18<br>(96.32 – 98.53)
**HS**	10.16 ± 0.88<br>(4.68 – 15.83)	96.72 ± 0.52<br>(91.19 – 98.78)
